# CSTF3 contributes to platinum resistance in ovarian cancer through alternative polyadenylation of lncRNA NEAT1 and generating the short isoform NEAT1_1

**DOI:** 10.1038/s41419-024-06816-1

**Published:** 2024-06-19

**Authors:** Xin Luo, Qinglv Wei, Xiaoyan Jiang, Ningxuan Chen, Xinzhao Zuo, Hongyan Zhao, Yujiao Liu, Xiaoyi Liu, Lingcui Xie, Yu Yang, Tao Liu, Ping Yi, Jing Xu

**Affiliations:** 1 Department of Obstetrics and Gynecology, The Third Affiliated Hospital of Chongqing Medical University, Chongqing, China; 2 Chongqing Key Laboratory of Child Infection and Immunity, Children’s Hospital of Chongqing Medical University, Chongqing, China; 3 School of Basic Medicine, Hubei University of Medicine, Shiyan, Hubei China

**Keywords:** Cancer epigenetics, Predictive markers, Cancer therapeutic resistance, Gene silencing, Cell signalling

## Abstract

Platinum-based chemotherapy is the standard postoperative adjuvant treatment for ovarian cancer (OC). Despite the initial response to chemotherapy, 85% of advanced OC patients will have recurrent disease. Relapsed disease and platinum resistance are the major causes of death in OC patients. In this study, we compared the global regulation of alternative polyadenylation (APA) in platinum-resistant and platinum-sensitive tissues of OC patients by analyzing a set of single-cell RNA sequencing (scRNA-seq) data from public databases and found that platinum-resistant patients exhibited global 3’ untranslated region (UTR) shortening due to the different usage of polyadenylation sites (PASs). The APA regulator CSTF3 was the most significantly upregulated gene in epithelial cells of platinum-resistant OC. CSTF3 knockdown increased the sensitivity of OC cells to platinum. The lncRNA NEAT1 has two isoforms, short (NEAT1_1) and long (NEAT1_2) transcript, because of the APA processing in 3’UTR. We found that CSTF3 knockdown reduced the usage of NEAT1 proximal PAS to lengthen the transcript and facilitate the expression of NEAT1_2. Downregulation of the expression of NEAT1 (NEAT1_1/_2), but not only NEAT1_2, also increased the sensitivity of OC cells to platinum. Overexpressed NEAT1_1 reversed the platinum resistance of OC cells after knocking down CSTF3 expression. Furthermore, downregulated expression of CSTF3 and NEAT1_1, rather than NEAT1_2, was positively correlated with inactivation of the PI3K/AKT/mTOR pathway in OC cells. Together, our findings revealed a novel mechanism of APA regulation in platinum-resistant OC. CSTF3 directly bound downstream of the NEAT1 proximal PAS to generate the short isoform NEAT1_1 and was conducive to platinum resistance, which provides a potential biomarker and therapeutic strategy for platinum-resistant OC patients.

## Introduction

Ovarian cancer (OC) is the most lethal gynecological malignancy, with ~70% of diagnosed patients in advanced stages and a 5-year survival rate of below 30% for advanced patients [1]. The standard treatment for OC is debulking surgery combined with postoperative chemotherapy [2]. Platinum is the first-line chemotherapeutic agent for OC patients, and most patients respond well to initial chemotherapy [3, 4]. However, the majority of advanced OC patients develop platinum resistance, which leads to poor prognosis and high mortality [5]. Platinum resistance has become a major obstacle to the effective treatment of OC [6]. Therefore, understanding the molecular mechanism of platinum resistance and exploring the significant biomarkers for patients are urgent to improve OC outcomes.

Platinum resistance is a complex process that has been linked to epigenetic modifications, such as DNA and RNA methylation, histone modification, and regulation of noncoding RNAs [7, 8]. Recently, alternative polyadenylation (APA) has been recognized as an important regulator of epigenetic mechanisms [9]. APA is an essential RNA processing that generates mature RNA from precursor RNA (pre-RNA), including cleavage and polyadenylation [10]. More than 70% of human genes contain multiple polyadenylation sites (PASs) in their pre-RNA, and different usage of PAS could produce multiple RNA transcript isoforms with different mRNA coding sequences or lengths of RNA 3’ untranslated regions (UTRs) [11]. Therefore, APA processing contributes to the diversity of transcriptomes and proteomes, which affects RNA stability, transport, translation efficiency and protein localization [12]. Recent studies have shown that APA is widely involved in diverse physiological and pathological conditions, such as cell proliferation, differentiation, neural activation, and immune and stress responses [13–15]. Dysfunctional APA has been related to various cancers and other diseases [16, 17]. Among cancers, widespread shortening of 3′UTRs has been discovered in transcripts of many tumors compared with normal tissues or cells, including OC [18, 19]. Additionally, platinum-sensitive and platinum-resistant OC cells have shown distinct APA patterns [20]. However, the key regulator of APA and the molecular pathway for platinum resistance in OC have not been completely investigated.

In mammals, APA is regulated by the cleavage and polyadenylation complex (CPA), which is composed of four protein subcomplexes: cleavage and polyadenylation specific factors (CPSFs), cleavage stimulation factors (CSTFs), cleavage factor I mammalian (CFIm) and cleavage factor II mammalian (CFIIm), accompanied by numerous subunits [9]. In general, the APA reaction is controlled by distinct sequence elements, including the upstream A[A/U]UAAA hexamer and the downstream GU-/U-rich region of the PAS site [21]. These two sequences are recognized by the CPSFs and the CSTFs respectively [21]. CSTFs, comprising CSTF1 (CstF-50), CSTF2 (CstF-64) and CSTF3 (CstF-77), play a signaling role in APA processing by binding the GU-/U-rich region and contacting CPSFs [22]. As the largest factor of the CSTFs, CSTF3 acts as a bridge between CSTF1 and CSTF2 and interacts with CPSF complexes [23]. Significantly, disturbance of CSTF3 expression affects global mRNA 3′UTR length, protein expression and cell functions, which have been closely associated with the chemotherapy-sensitivity and progression of some cancers [22, 24]. For example, the retained intron (RI) in CSTF3 was an independent prognostic indicator for both overall survival (OS) and disease-free survival (DFS) in a colorectal cancer (CRC) cohort, and it could directly change the 3′UTR of nucleic acid sequences and affect the major progression of CRC [25]. CSTF3 was upregulated in triple-negative breast cancer (TNBC) cells, and CSTF3 promoted the proliferation of TNBC by inducing APA of NRAS and c-JUN [26]. In addition, CSTF3 was upregulated in 5-fluorouracil (5-FU)-sensitive gastric cancer (GC) cell lines and could be used as a biomarker of 5-FU for GC patients [27]. However, the role of CSTF3 in the formation and progression of OC remains elusive, especially in platinum resistance OC.

In this study, we found that the APA regulator CSTF3 was upregulated in platinum-resistant OC tissues and cells. Our functional study revealed that CSTF3 acts as an oncogene and promotes platinum resistance by activating the PI3K/AKT/mTOR signaling pathway in OC cells. Molecular mechanism exploration indicated that CSTF3 directly binds downstream of the NEAT1 proximal PAS and leads to the generation of the short transcript NEAT1_1, which contributes to platinum resistance in OC cells. In addition, the platinum resistance of OC was independent of the formation of paraspeckles.

## Results

### APA processing is associated with platinum resistance in OC, and the key regulator CSTF3 is highly expressed in platinum-resistant OC tissues

**Fig. 1:**
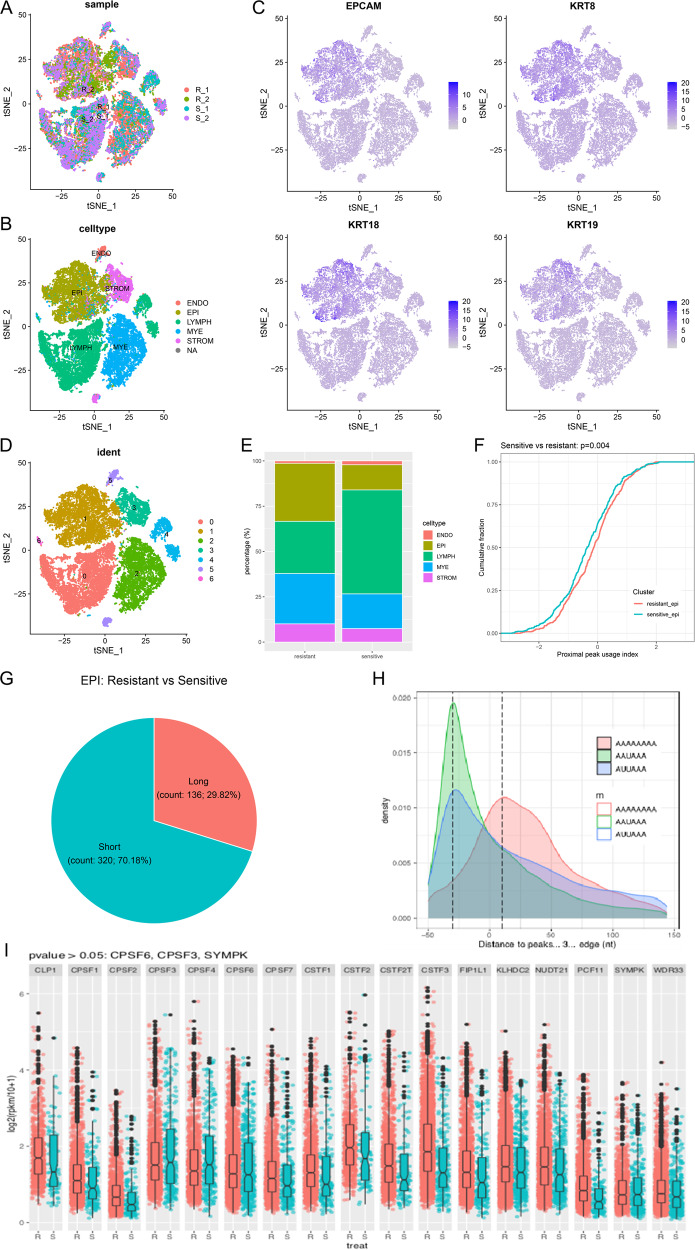
ScRNA-seq exploratory data analysis for platinum-resistant and sensitive OC tissues. **A** Composition of the four patient scRNA-seq datasets by tSNE plot. **B** Individual tumor cells were assigned to five cell types. **C** Annotation validation of epithelial cells (EPI) using the highly expressed epithelial marker genes EPCAM, KRT8, KRT18, and KRT19. **D** Cells were assigned to seven types based on the expression of variable genes and visualized using tSNE. **E** The proportion of cell types for platinum-resistant and platinum-sensitive patients. **F** The proximal peak usage index of platinum-resistant and platinum-sensitive epithelial cells. **G** Transcripts with significant 3 ‘UTR length changes between resistant and sensitive epithelial cells. **H** The distribution of APA peaks. **I** Differential expression analysis of APA primary regulators in platinum-resistant epithelial cells compared with sensitive cells.

To investigate whether APA processing is involved in platinum resistance in OC, we downloaded scRNA-seq data of platinum-resistant OC tissues from the GEO database, including 2 platinum-resistant and 2 platinum-sensitive OC tissues. After homogenization and integration, t-Distributed Stochastic Neighboring Embedding (t-SNE) and Uniform Manifold Approximation and Projection (UMAP) dimensionality reductions were performed, and different sources of cells were distributed in all populations, indicating successful integration (Fig. 1A and Supplementary Fig. 1A, B). Then, the cell type was annotated via marker gene expression, and five major cell type populations were acquired: epithelial, endothelial, lymphocyte, myeloid, and stromal, according to previous research (Fig. 1B and Supplementary Fig. 1C) [28]. The epithelial marker genes EPCAM, KRT8, KRT18, and KRT19 were highly expressed in epithelial cells (Fig. 1C and Supplementary Fig. 1D), and the proportion of epithelial cells was significantly increased in platinum-resistant tissues compared with platinum-sensitive tissues (Fig. 1E). In addition, we divided all cells into 7 clusters using the Seurat package (Fig. 1D and Supplementary Fig. 1E), and the specific genes were expressed in each cluster, such as cluster 0, defined lymphocytes, containing CD6/CD27 genes, and cluster 1, defined epithelial cells, specifically expressing the KRT8 gene (Supplementary Fig. 1F). We followed up with sequencing analysis of individual epithelial cancer cells and explored whether epithelial cells have different APA phenotypes between platinum-resistant and platinum-sensitive OC tissues. platinum-resistant patients had substantial differences in the 3’ UTR length of transcripts in comparison to platinum-sensitive patients, and the resistant cells were more inclined to use proximal PAS (Fig. 1F). There were 320 transcripts with shortened and 132 transcripts with lengthened in platinum resistance (Fig. 1G). The obtained peaks were enriched in the APA conserved sequences AAUAAA and AUUAAA, which were mainly distributed at the 3’ end of the transcripts (Fig. 1H). Since APA processing is regulated by a series of special factors, we analyzed the mRNA expression of APA core regulators in epithelial cells and found that most of them were upregulated in platinum-resistant tissues. Among these, the expression of CSTF3 was the most significantly upregulated (Fig. 1I). These results suggested that platinum-resistant tissues exhibited APA and might be regulated by core regulators in OC epithelial cells.

### CSTF3 facilitates platinum resistance of OC cells in vitro and in vivo

**Fig. 2:**
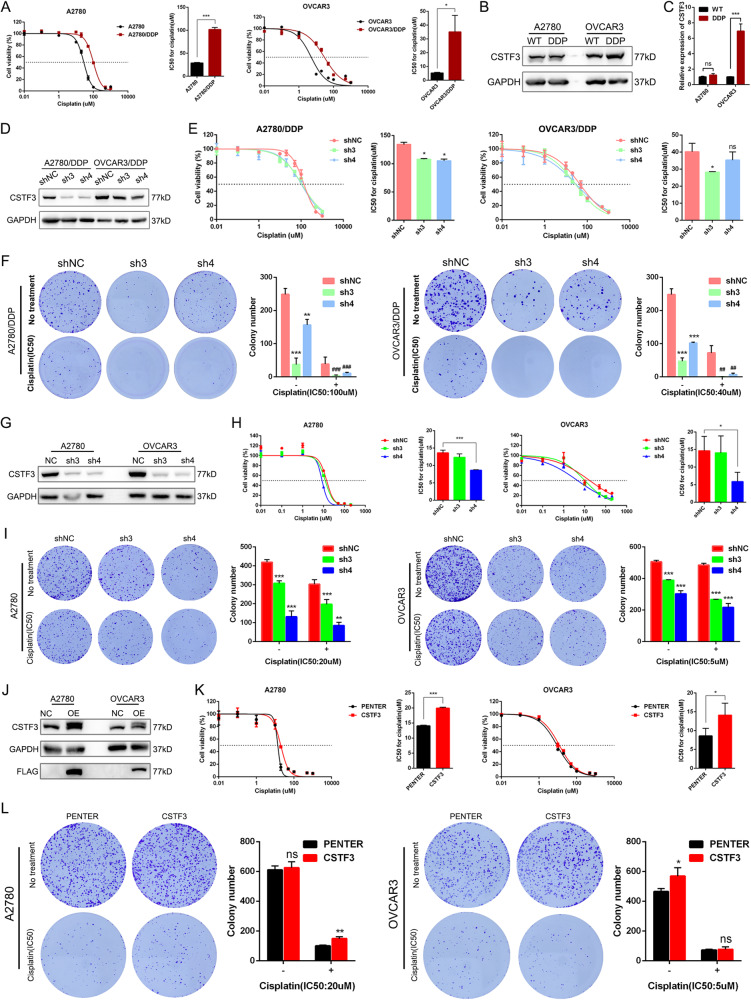
CSTF3 facilitated platinum resistance in OC cells in vitro. **A** Construction of platinum-resistant OC cell lines A2780-DDP and OVCAR3-DDP cells. Western blot (**B**) and RT‒qPCR (**C**) were performed to detect the expression of CSTF3 in A2780-DDP, OVCAR3-DDP and corresponding parental cells. **D** CSTF3 protein was measured to confirm the knockdown efficiency in A2780-DDP and OVCAR3-DDP cells. **E** Cell viability was evaluated to measure the IC50 of platinum after knockdown of CSTF3 and treatment with gradient concentrations of platinum in A2780-DDP and OVCAR3-DDP cells. **F** A2780-DDP and OVCAR3-DDP cells were treated with 100 μM and 40 μM platinum for 6 h respectively. Colony formation assay was performed with control and CSTF3 knockdown A2780-DDP and OVCAR3-DDP cells treated with or without platinum. **G** Western blot was performed to verify CSTF3 knockdown efficiency in WT A2780 and OVCAR3 cells. **H** A2780 and OVCAR3 cells with CSTF3 knockdown were treated with different concentrations of platinum, and cell survival was determined. **I** A2780 and OVCAR3 cells were treated with 20 μM and 5 μM platinum for 6 h respectively. Colony formation assays were performed in A2780 and OVCAR3 cells treated with or without platinum after CSTF3 knockdown. **J** FLAG-NC and FLAG-CSTF3 were stably transfected into A2780 and OVCAR3 cells. Then, cell viability curves (**K**) and colony formation assays (**L**) were performed to measure the sensitivity of cells to platinum after overexpression of CSTF3. *P < 0.05, **P < 0.01, ***P < 0.001 vs. shNC.

To deeply study platinum resistance in OC, we established two platinum-resistant OC cell lines, A2780 and OVCAR3, according to our method. We first examined the resistance index (RI) of A2780‐DDP cells >3 and OVCAR3‐DDP cells >5, suggesting that the construction of platinum‐resistant OC cell lines was successful (Fig. 2A). These models were used for subsequent in vitro and in vivo experiments. The above scRNA-seq analysis identified higher expression of CSTF3 in platinum-resistant tissues than in platinum-sensitive OC tissues. Using western blot and RT-qPCR, we confirmed that the expression of CSTF3 protein and mRNA was higher in platinum‐resistant OVCAR3 cells (OVCAR3‐DDP) than in parental OVCAR3 cells (Fig. 2B, C). We speculated that high expression of CSTF3 could confer platinum resistance in OC cells. To evaluate the effect of CSTF3 on platinum resistance, we constructed two shRNAs to inhibit the expression of CSTF3 in parental and resistant OC cells, and the cells were treated with or without platinum after stable knockdown (Fig. 2D, G and Supplementary Fig. 2). Using the cell viability assay, the IC50 was examined after exposure to gradient platinum for 48 h, and knockdown of CSTF3 in platinum-resistant or parental OC cells significantly decreased the IC50 of platinum (Fig. 2E, H). Moreover, using a colony formation assay, we found that knockdown of CSTF3 decreased the number of colonies in resistant and parental OC cells compared with the empty vector. After treatment with platinum, the degree of decrease in colony formation was more pronounced in the knockdown groups, which also indicated that CSTF3 knockdown dramatically increased platinum sensitivity in platinum-resistant and parental OC cells (Fig. 2F, I). Furthermore, we found that knockdown of CSTF3 significantly inhibited the proliferation of A2780 and OVCAR3 cells through CCK-8 and colony formation assays (Supplementary Fig. 2B, C). Both the A2780 and OVCAR3 cell lines were treated with platinum for 24 h to induce DNA damage, and the flow cytometry results also showed a higher proportion of apoptotic cells in the CSTF3 knockdown group than in the negative control group (Supplementary Fig. 2D). In contrast, we upregulated the expression of CSTF3 in parental A2780 and OVCAR3 cells (Fig. 2J). Similarly, using cell viability and colony formation assays, the results showed that CSTF3 overexpression significantly enhanced the IC50 value and ability of proliferation in A2780 and OVCAR3 cells treated with or without platinum (Fig. 2K, L).

**Fig. 3:**
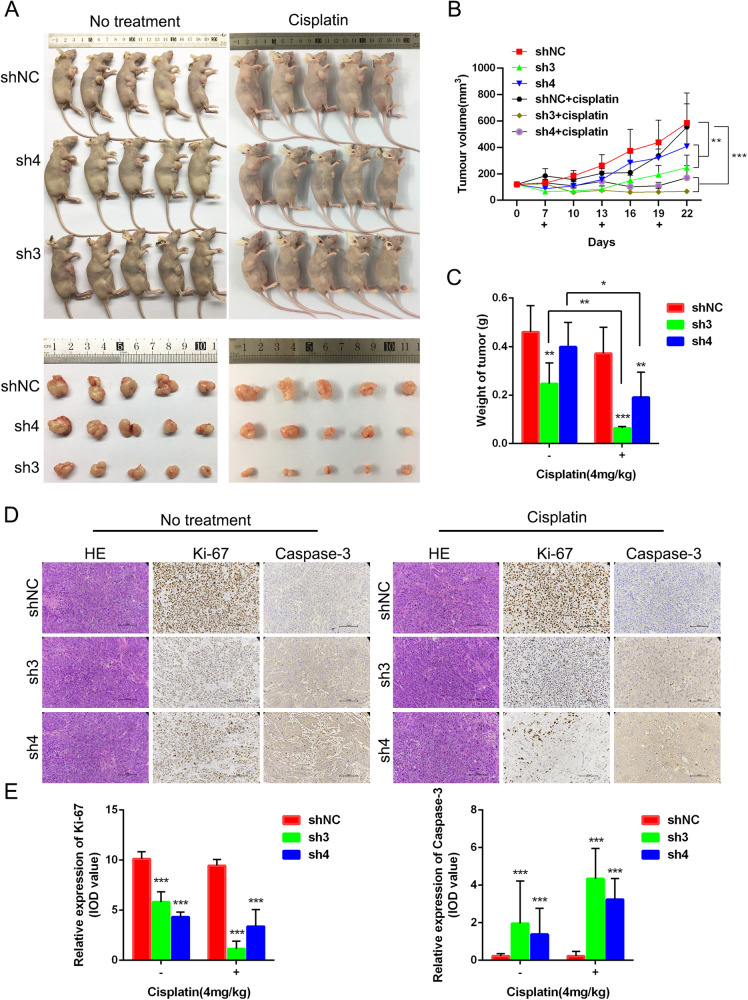
CSTF3 facilitated platinum resistance in OC cells in vivo. OVCAR3 cells transfected with CSTF3 knockdown or negative control constructs were injected subcutaneously into BALB/c nude mice. The xenografted mice were treated with or without DDP (4 mg/kg) through intraperitoneal injection once every 6 days. **A** Photo of representative tumors from six groups of xenografted nude mice on the 22nd day. **B** The growth of tumor volumes was monitored every 3 days beginning on the 7th day. **C** Tumor weights are presented as the mean ± SD from five mice per group. **D**, **E** HE staining was performed to evaluate tissue morphology, and IHC was performed to visualize Ki-67- and Caspase-3-positive staining in xenografted tumors. *P < 0.05, **P < 0.01, ***P < 0.001 vs. shNC.

Furthermore, the role of CSTF3 in platinum resistance was investigated using the xenograft model. Female nude mice were subcutaneously injected with CSTF3 knockdown OVCAR3 cells and treated with saline or DDP (4 mg/kg). CSTF3 knockdown significantly inhibited the growth of xenograft tumors and dramatically reduced the volume and weight of tumors; after platinum treatment, the volume and weight of tumors were more significantly suppressed in the CSTF3 knockdown groups than in the negative control group (Fig. 3A–C). Subsequently, the morphological examination of tumors was performed by HE staining, and the expression of the proliferative protein Ki-67 and the apoptotic protein Caspase-3 in xenografts was evaluated by IHC (Fig. 3D). The statistical results showed that knockdown of CSTF3 inhibited the expression of Ki-67 and enhanced the expression of Caspase-3 in xenografts, and knockdown of CSTF3 caused more significant differential expression under platinum treatment compared with saline treatment (Fig. 3E). Together, these data suggested that downregulation of CSTF3 markedly enhanced the platinum sensitivity of OC and that overexpression of CSTF3 contributed to platinum resistance in OC cells.

### CSTF3 regulates the length of the 3’UTR of lncRNA NEAT1 in OC cells

**Fig. 4:**
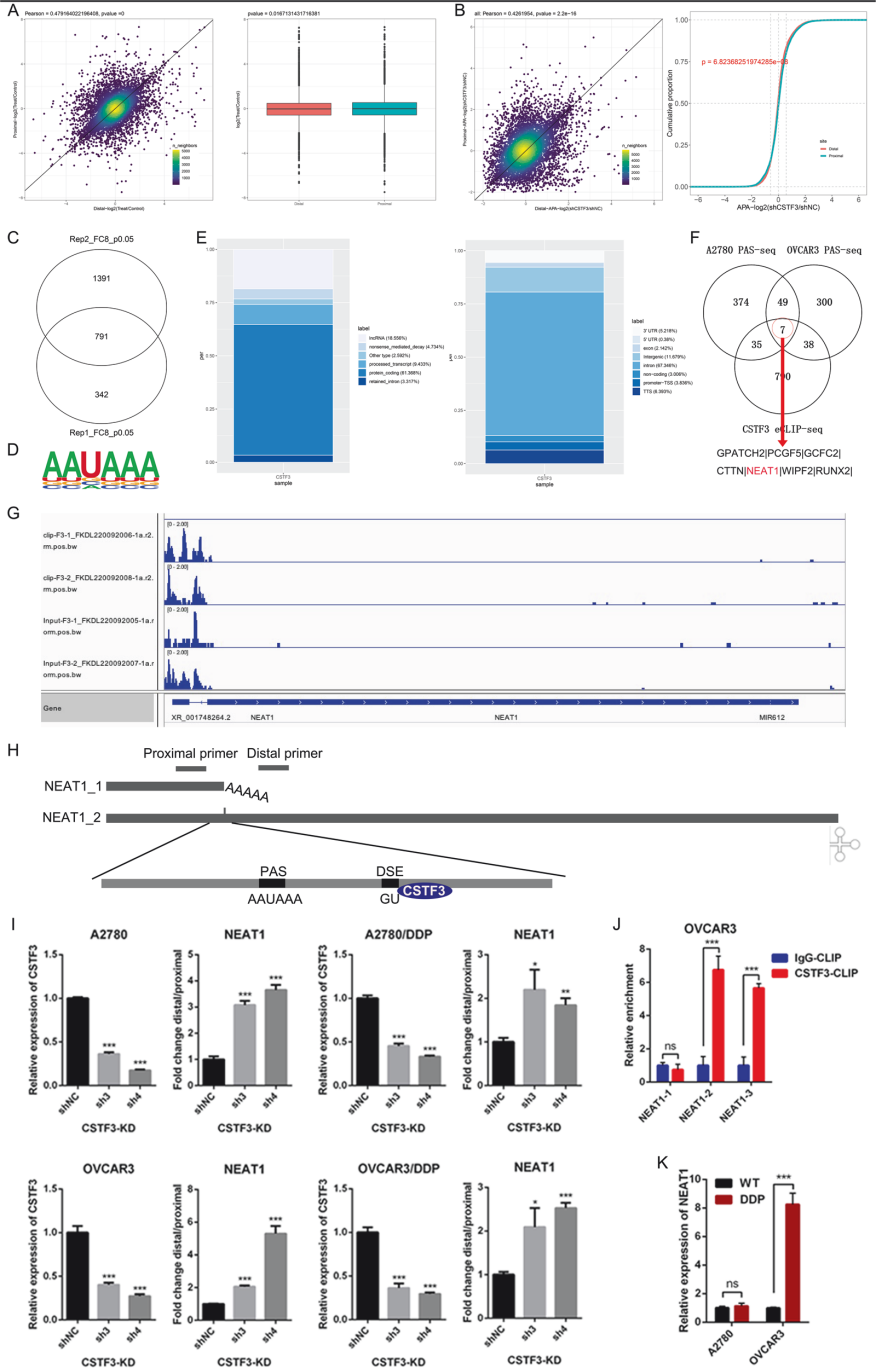
CSTF3 bound and regulated the length of the 3’UTR of lncRNA NEAT1 in OC cells. **A** CSTF3 knockdown induced an APA shift of target genes in A2780 cells, left: scatterplot of the 3’UTR alteration in CSTF3 knockdown cells when compared with the control cells, right: the usage of proximal and distal PAS in CSTF3 knockdown cells. **B** Detection of APA events in CSTF3 knockdown OVCAR3 cells, combining 3’UTR alteration and usage of PAS. **C** Filtering out the directly bound target genes of CSTF3 by eCLIP-seq. **D** The consensus sequences of CSTF3 binding sites detected by HOMER motif analysis with eCLIP-seq data. **E** Distribution of CSTF3-targeted transcript types (left) and CSTF3 binding sites within different regions (right) as identified through eCLIP-seq. **F** The Venn diagram screened the candidate target genes using CSTF3 eCLIP-seq, A2780 and OVCAR3 PAS-seq data. **G** IGV genome browser showing the PAS usage of the NEAT1 3′UTR by eCLIP-seq. **H** Schematic diagram for primer design of short and long NEAT1 transcripts to validate the NEAT1 APA regulation by CSTF3. **I** Histogram showing the relative expression of the NEAT1 isoform with the distal PAS (dPAS) relative to that with the proximal PAS (pPAS) when CSTF3 was downregulated in A2780, A2780-DDP, OVCAR3 and OVCAR3-DDP cells compared with the negative control. **J** CLIP-qPCR detected the interaction between CSTF3 and NEAT1. **K** RT-qPCR was used to detect the relative expression of NEAT1 in A2780-DDP, OVCAR3-DDP and corresponding parental cells. *P < 0.05, **P < 0.01, ***P < 0.001.

To elucidate the mechanisms of platinum resistance and identify potential targets of CSTF3 in OC cells, we performed PAS-seq on CSTF3 knockdown and control cells, and the quantified PAS usage and length variation of transcripts were analyzed (Supplementary Fig. 3A, B). Through APA analysis of PAS-seq data, we identified 293 transcripts with shortened 3’UTRs and 172 transcripts with lengthened 3’UTRs upon downregulation of CSTF3 in A2780 cells (Fig. 4A). Meanwhile, 148 transcripts with shortened 3’UTRs and 247 transcripts with lengthened 3’UTRs were identified following CSTF3 knockdown in OVCAR3 cells (Fig. 4B). To further investigate the direct binding sequences of CSTF3 in OC cells, we performed eCLIP-seq and identified 791 transcripts as target genes (Fig. 4C and Supplementary Fig. 3C), and the AAUAAA motif was highly enriched in the CSTF3 binding site of OVACR3 cells (Fig. 4D). These motifs were mainly located in protein-coding genes or intronic regions (Fig. 4E). Then, the PAS-seq gene sets were intersected with the eCLIP-seq genes, and 7 genes were screened out as candidate target genes (Fig. 4F). These genes contained GPATCH2, PCGF5, GCFC2, CTTN, NEAT1, WIPF2, RUNX2. The NEAT1 is an important long non-coding RNA (lncRNA), which is widely involved in a variety of cellular biological processes [29]. Aligned with the NCBI and UCSC genome browser databases, we found that lncRNA NEAT1 had two isoforms with different 3’UTR lengths. According to previous literature reports, generation of the short (NEAT1_1) and long (NEAT1_2) splice variants of NEAT1 was related to APA regulation through the HNRNPK and CPSF6-NUDT21 proteins [30]. However, the specific function of NEAT1_1 and NEAT1_2 in platinum-resistant OC and the APA regulation of NEAT1 by CSTF3 have not been reported in the literature. The integrative genomics viewer (IGV) map from PAS-seq and eCLIP-seq also showed that the usage of the NEAT1 proximal PAS was significantly decreased after CSTF3 knockdown, and CSTF3 could bind NEAT1 loci at the 3’UTR compared with the input signal (Fig. 4G). Thus, we selected NEAT1 as an important target for further research. The distinct primers for recognizing NEAT1 (NEAT1_1/_2) and the long isoform (NEAT1_2) of NEAT1 were designed in Supplementary Table 2 and a schematic diagram (Fig. 4H). RT-qPCR experiments were used to detect the expression of NEAT1 and NEAT1_2 in parental and platinum-resistant OC cells transfected with shRNAs targeting CSTF3 or control. We observed that the usage of the proximal PAS was decreased with CSTF3 knockdown in OC cells, which led to increased the ratio of the long variant NEAT1_2 to NEAT1 (Fig. 4I). More significantly, according to the analysis of eCLIP-seq data, the binding sequences are located downstream of the NEAT1 proximal PAS. Next, we designed three pairs of primers (NEAT1-1, NEAT1-2 and NEAT1-3) near the CSTF3 binding site region (Table S2). Using CSTF3 eCLIP-qPCR (Supplementary Fig. 3C), the amplified sequences of NEAT1-2 (binding site) and NEAT1-3 (downstream) primers but not NEAT1-1 (upstream) were directly bound by CSTF3 in OVCAR3 cells compared with the negative control IgG antibody (Fig. 4J). In addition, NEAT1 was found to be expressed at significantly higher levels in platinum-resistant OVCAR3 cells than in parental OVCAR3 cells (Fig. 4K). Overall, CSTF3 could directly bind downstream of the NEAT1 proximal PAS and promote proximal PAS choice to increase the expression of the short transcript NEAT1_1 and decrease the generation of the long transcript NEAT1_2, which might be a major APA event for OC platinum resistance.

### The short 3’UTR isoform of NEAT1 promotes platinum resistance in OC cells

**Fig. 5:**
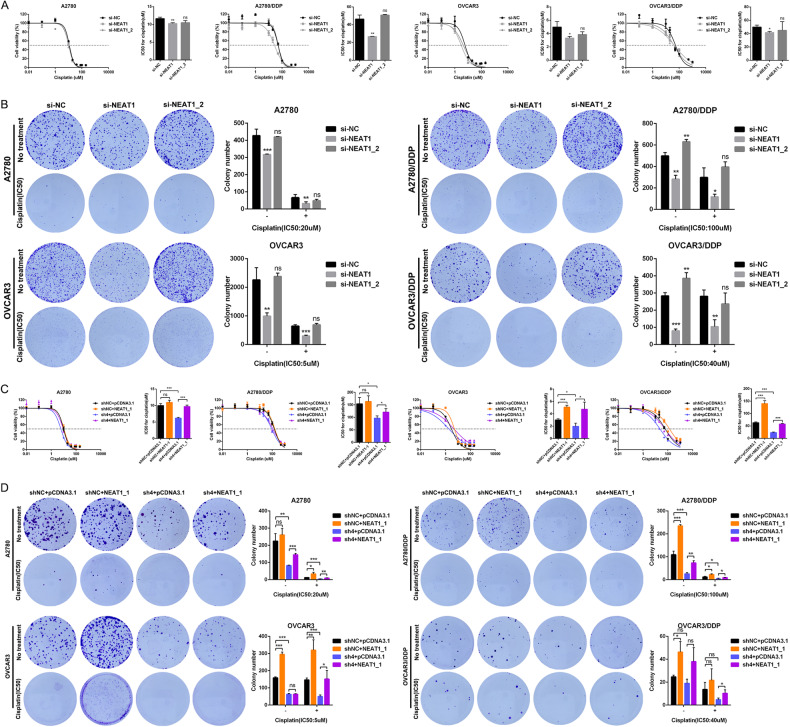
The short 3’UTR isoform of NEAT1 promotes platinum resistance in OC cells. **A** The IC50 values were evaluated when silencing the expression of NEAT1 and NEAT1_2 in A2780, OVCAR3 and corresponding platinum-resistant cells compared with the negative control. **B** A2780, OVCAR3, A2780-DDP and OVCAR3-DDP cells were treated with 20 μM, 5 μM, 100 μM and 40 μM platinum for 6 h respectively. Colony formation assay was performed to measure the sensitivity of the drug when silencing the expression of NEAT1 and NEAT1_2 in A2780, OVCAR3 and corresponding platinum-resistant cells treated with or without platinum. **C**, **D** The IC50 values and colony formation were evaluated when NEAT1_1 was overexpressed in A2780, OVCAR3 and platinum-resistant cells with CSTF3 knockdown treated with platinum or not. *P < 0.05, **P < 0.01, ***P < 0.001. ns not significant.

To understand the function of distinct NEAT1 transcript isoforms in OC platinum resistance, siRNAs targeting NEAT1 (NEAT1_1/_2) and NEAT1_2 were transfected into A2780, OVCAR3 and corresponding platinum-resistant cells (Supplementary Fig. 4A). We observed that the expression of the long isoform NEAT1_2 accounted for only ~1/10 of the total NEAT1 in these OC cells (Supplementary Fig. 4A). Cell viability and colony formation assays were performed to examine the sensitivity of OC cells to platinum. The results showed that silencing NEAT1 dramatically decreased the IC50 value against platinum in A2780, OVCAR3 and corresponding platinum-resistant cells compared with the negative control cells, while the IC50 had no significant change when only silencing NEAT1_2 in cells (Fig. 5A). Based on the colony formation assay, we further observed that silencing the expression of NEAT1 inhibited the proliferation of A2780, OVCAR3 and platinum-resistant cells, and the degree of inhibition was more pronounced after platinum treatment, while no significant difference was observed after only silencing NEAT1_2 compared with the negative control (Fig. 5B). These findings suggested that the short NEAT1 transcript NEAT1_1 might play a crucial role in mediating platinum resistance in OC cells. To verify this speculation, a rescue experiment was performed in which NEAT1_1 was overexpressed in A2780, OVCAR3 and platinum-resistant cells with CSTF3 knockdown (Supplementary Fig. 4B). The results showed that CSTF3 knockdown substantially decreased the IC50 value of platinum in A2780, OVCAR3 and corresponding platinum-resistant cells, and overexpression of NEAT1_1 reversed the decreased IC50 value of platinum (Fig. 5C). Then, the colony formation assay also found that CSTF3 knockdown suppressed the proliferation of A2780, OVCAR3 and corresponding platinum-resistant cells with or without platinum treatment. Overexpression of NEAT1_1 could enhance the proliferation of parental or platinum-resistant OC cells and reverse proliferation ability after CSTF3 knockdown (Fig. 5D). Collectively, these results showed that NEAT1_2 was irrelevant to the platinum resistance of OC cells, while the generation of the short 3’UTR isoform NEAT1_1, regulated by CSTF3, was sufficient to promote platinum resistance in OC cells.

### NEAT1 isoforms contribute to platinum resistance through the paraspeckle-independent pathway in OC cells

**Fig. 6:**
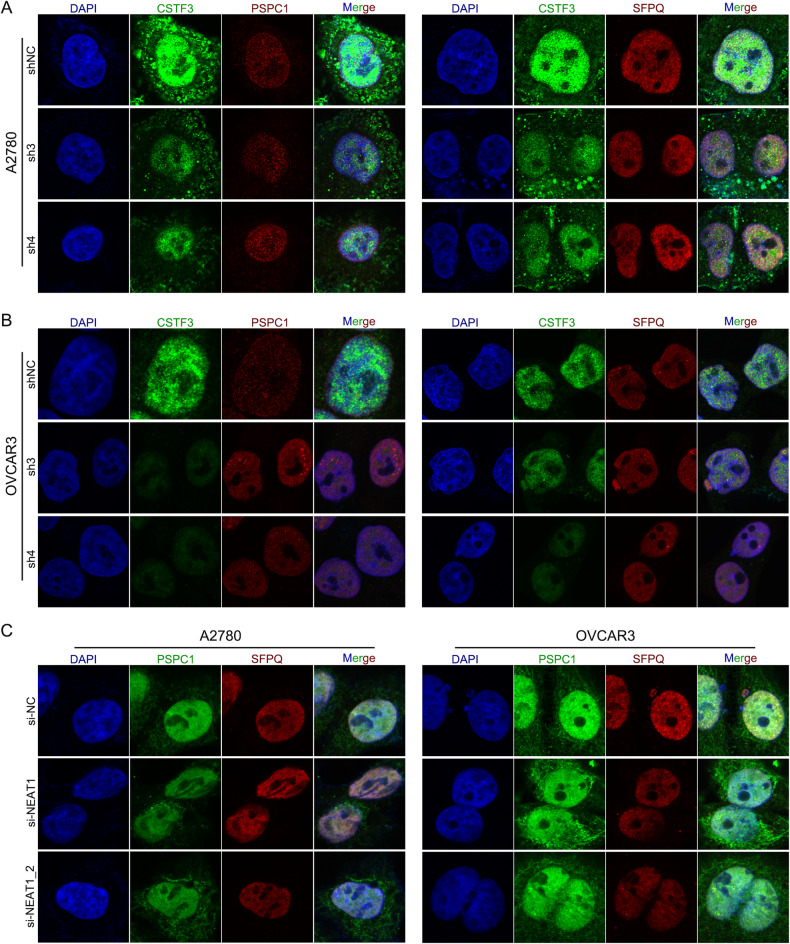
The change of paraspeckles formation in A2780 and OVCAR3 cells when downregulating the expression of CSTF3, NEAT1 or NEAT1_2. Immunofluorescence assays were performed with CSTF3 knockdown in A2780 (**A**) and OVCAR3 (**B**) cells, and images of DAPI (blue), CSTF3 (green), PSPC1 (red, left) and SFPQ (red, right) were obtained. **C** Immunofluorescence of A2780 and OVCAR3 cells transfected with si-NEAT1, si-NEAT1_2 and the negative control. DAPI (blue), PSPC1 (green) and SFPQ (red) were detected.

Based on previous studies, paraspeckles are a type of subnuclear body built on the lncRNA NEAT1 that is involved in diverse physiological processes, including cell differentiation, stress responses, neurodegeneration, and cancer progression [29]. Because NEAT1 is necessary for paraspeckle assembly, it is possible that imbalance of two distinct NEAT1 transcripts (NEAT1_1 and NEAT1_2) leads to the disruption of paraspeckles [31]. Additionally, paraspeckles are membraneless nuclear organelles that are dynamically regulated by NEAT1 and several paraspeckle proteins, including NONO, PSPC1, SFPQ, and hnRNPs [32]. To investigate whether the regulation of NEAT1 APA by CSTF3 affects the assembly of paraspeckles and whether the formation of paraspeckles affects the progression of platinum resistance in OC. Here, we downregulated the expression of CSTF3, NEAT1 and NEAT1_2 in A2780 and OVCAR3 cells, and the essential paraspeckle proteins PSPC1 and SFPQ were examined using an immunofluorescence assay. As shown in Fig. 6A, B, there was no significant difference in the assembly of PSPC1 and SFPQ when downregulating the expression of CSTF3 in A2780 and OVCAR3 cells. However, when the expression of NEAT1 and NEAT1_2 was downregulated, the aggregation degree of PSPC1 and SFPQ was significantly reduced in A2780 and OVCAR3 cells, which indicated a decrease in the ability to form paraspeckles (Fig. 6C). Previous studies have demonstrated that the transcript NEAT1_2 but not NEAT1_1 is indispensable for paraspeckle formation and that NEAT1_2 acts as the architectural backbone of paraspeckles [30]. Loss of function of NEAT1 and NEAT1_2 leads to a failure of paraspeckle formation [33]. which is consistent with our above observations. Therefore, based on these findings and the phenotype of platinum resistance after silencing NEAT1_2, we suspect that the formation and function of paraspeckles are not related to platinum resistance in OC cells.

### CSTF3 and NEAT1_1 regulate PI3K/AKT pathway-mediated platinum resistance in OC cells

**Fig. 7:**
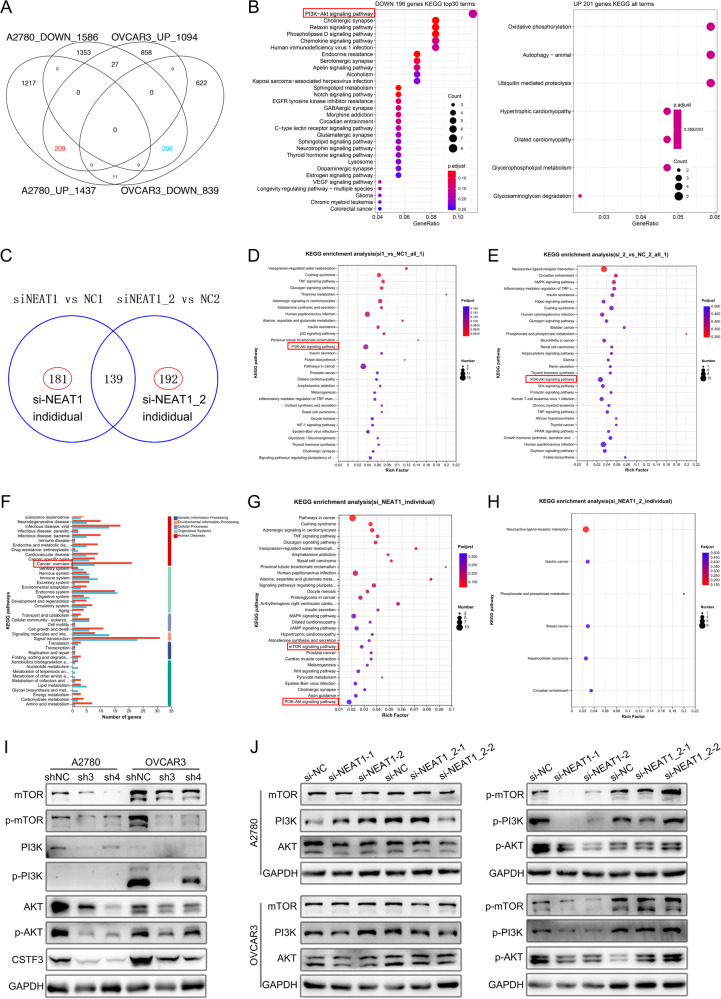
CSTF3 and NEAT1_1 regulate the PI3K/AKT pathway. **A** Overlap of upregulated and downregulated genes in A2780 and OVCAR3 cells when CSTF3 was knocked down. **B** KEGG enrichment analysis for overlap of upregulated and downregulated genes. **C** Overlap of differentially expressed genes (DEGs) in OVCAR3 cells when NEAT1 and NEAT1_2 were silenced. **D** KEGG analysis of DEGs for silencing NEAT1. **E** KEGG analysis of DEGs for silencing NEAT1_2. **F** Histogram of KEGG analysis for the independent DEGs list with NEAT1 and NEAT1_2 silenced. **G** KEGG enrichment analysis of the individual DEGs list with NEAT1 silenced. **H** KEGG enrichment analysis of the individual DEGs list with NEAT1_2 silenced. **I** The levels of PI3K/AKT/mTOR pathway-related proteins were detected by western blot when CSTF3 was downregulated in A2780 and OVCAR3 cells. **J** Western blot was used to measure the levels of PI3K/AKT/mTOR pathway-related proteins with NEAT1 or NEAT1_2 siRNA transfected into A2780 and OVCAR3 cells.

To further explore how CSTF3-modulated NEAT1 APA contributes to platinum resistance, we first analyzed the differentially expressed genes (DEGs) of CSTF3 knockdown based on the PAS-seq data in A2780 and OVCAR3 cells (Supplementary Fig. 3D, E). The KEGG enrichment analysis of DEGs showed that these downregulated genes were involved in many cancer-related pathways, such as the PI3K/AKT pathway, Hippo pathway, Notch pathway and p53 signaling pathway (Supplementary Fig. 3D, E). By crossing the upregulated and downregulated genes (Fig. 7A), we found that the overlap of downregulated genes was also enriched in multiple cancer-related pathways, with the PI3K/AKT pathway ranking first (Fig. 7B). Second, we executed RNA-seq on OVCAR3 cells in which NEAT1 and NEAT1_2 were successfully downregulated. The differential genes with downregulation of NEAT1 and NEAT1_2 were assessed (Supplementary Fig. 5A–C). When NEAT1 was silenced in OVCAR3 cells, GO enrichment analysis showed that the DEGs were participated in response to stimulus, catalytic activity and organelle part (Supplementary Fig. 5D), and KEGG analysis showed that DEGs were involved in the p53 pathway, PI3K/AKT pathway and pathways in cancer (Fig. 7D). When NEAT1_2 was silenced in OVCAR3 cells, GO and KEGG analyses showed that the DEGs were enriched in catalytic activity, cellular processes, cell death, the Hippo pathway, the PI3K/AKT pathway and the Wnt signaling pathway (Supplementary Fig. 5E and Fig. 7E). Meanwhile, we intersected the differential gene sets of silenced NEAT1 and NEAT1_2 and analyzed the differential genes outside the intersection individually (Fig. 7C). There were 181 independent DEGs from NEAT1-silenced cells and 192 independent DEGs from NEAT1_2-silenced cells (Fig. 7C). Histogram of GO and KEGG analysis showed that independent DEGs of silenced NEAT1 were more significantly enriched in organelle, catalytic activity, cancer, cell growth and death, signal transduction, and amino acid metabolism compared to the independent DEGs of silenced NEAT1_2 (Fig. 7F and Supplementary Fig. 5F). Furthermore, we observed that the independent DEGs of silenced NEAT1 were involved in many cancer pathways, the MAPK pathway, the mTOR pathway, the Wnt pathway and the PI3K/AKT pathway, while the independent DEGs of silenced NEAT1 were only enriched in gastric cancer, breast cancer and hepatocellular carcinoma (Fig. 7G, H). There was no obvious difference in GO enrichment between the independent DEGs of NEAT1 and NEAT1_2 (Supplementary Fig. 5G, H).

To verify possible downstream signaling mechanisms, proteins related to the PI3K/AKT/mTOR signaling pathway were observed by western blot. The results revealed that the expression of p-AKT, p-PI3K, p-mTOR, AKT, PI3K and mTOR was decreased in A2780 and OVCAR3 cells with CSTF3 knockdown compared to control cells (Fig. 7I). On the other hand, we found that p-AKT, p-PI3K and p-mTOR expression decreased significantly in A2780 and OVCAR3 cells transfected with si1-NEAT1 or si2-NEAT1, while there was no significant difference in si1-NEAT1_2 and si2-NEAT1_2 cells when compared with their corresponding control cells (Fig. 7J). AKT, PI3K and mTOR expression were not affected after silencing NEAT1 or NEAT1_2 in A2780 and OVCAR3 cells (Fig. 7J). In summary, these data confirmed that CSTF3 knockdown and NEAT1 silencing suppressed the activity of the PI3K/AKT/mTOR pathway in OC cells. Nevertheless, only silencing NEAT1_2 had no effect on the PI3K/AKT/mTOR pathway, and we deduced that CSTF3 regulates the APA processing of NEAT1 to generate the NEAT1_1 transcript in OC and further promote platinum resistance through the AKT/PI3K pathway.

## Discussion

Platinum-based chemotherapy is the standard first-line chemotherapy in the treatment of OC [34]. Primary OC patients are initially responsive to chemotherapy, but the majority of patients with advanced stages of OC develop recurrent disease because of platinum resistance [35]. These recurrent patients are classified as platinum-sensitive relapse (relapse >6 months following last platinum chemotherapy) and platinum-resistant relapse (relapse <6 months following initial platinum chemotherapy) [36]. Almost all recurrent OC patients will resist conventional chemotherapy early or later, which is associated with a high mortality rate and poor prognosis in patients [37]. Therefore, novel strategies and targets are urgently needed to improve the outcome for platinum-resistant patients.

Increasing evidence has shown that epigenetic alterations play an important role in OC platinum resistance [38]. Recently, several studies have indicated that APA resulting in alternative 3′UTRs affects epigenetic regulation [39]. However, it remains unknown whether the APA processing of genes is involved in OC platinum resistance. Herein, we observed an obvious difference in APA processing between platinum-resistant tissues and platinum-sensitive tissues of OC. Consistent with a previous study [20]. we found that compared with platinum-sensitive cells, platinum-resistant cancerous epithelial cells tended to express RNA transcripts with shortened 3′ UTRs. In mammals, the complexes CPSF, CSTF, CFIm and CFIIm are machinery responsible for APA [40]. CSTF3, belonging to the CSTF subcomplex, was the most significantly increased in platinum-resistant OC compared with other APA regulators. Hence, we selected CSTF3 to carry out the following exploration.

To date, relatively few reports have been acquired on CSTF3 for many diseases and cancer research. By constructing platinum-resistant OC cell lines and combining multiple phenotypic experiments in vitro and in vivo, we observed high expression of CSTF3 in platinum-resistant cells, and CSTF3 contributed to platinum-resistance of OC. Furthermore, we downregulated the expression of CSTF3 to determine whether CSTF3 affects the APA processing of target genes in OC cells. Conjoint analysis of PAS-seq and eCLIP-seq data identified a meaningful target gene lncRNA, NEAT1, and executed an eCLIP-qPCR assay to confirm NEAT1 as a direct binding gene of CSTF3.

NEAT1, a polyadenylated lncRNA, has been widely identified as an oncogene that promotes tumor formation and metastasis in various solid malignancies [41]. In OC, NEAT1 is more highly expressed in cancers than in normal tissues, and it facilitates tumorigenesis and progression, high expression of NEAT1 is correlated with unfavorable prognosis [42]. In particular, NEAT1 knockdown improved paclitaxel (PTX) sensitivity of OC by regulating the miR-194/ZEB1 axis, and NEAT1 knockdown enhanced platinum sensitivity in OC cells through miR-770-5p upregulation and PARP1 downregulation, but the function of NEAT1 different transcripts (NEAT1_1 and NEAT1_2) in mediating platinum resistance in OC is unclear [43, 44]. According to a previous study, NEAT1 is spliced by APA processing to generate short and long splice isoforms, NEAT1_1 and NEAT1_2 [33]. APA processing is regulated by the opposing actions of the HNRNPK protein and CFIm complex around the PAS of NEAT1, and downregulated expression of NUDT21 or CPSF6 prominently diminishes NEAT1_1 and simultaneously increases the NEAT1_2 leve [30]. In our study, we found that knockdown of CSTF3 also markedly reduced the ratio of NEAT1_1 to NEAT1_2 in OC cells, which was consistent with the PAS-seq results. CSTF3 could enhance proximal PAS usage of NEAT1 to generate the short variant by directly binding downstream of the PAS.

The two NEAT1 isoforms may have distinct or even opposite functions and have different locations in cells [45]. A previous report has shown that only NEAT1_2, but not NEAT1, is significantly correlated with poor overall survival of hepatocellular carcinoma (HCC) patients and promotes tumor development by mediating paraspeckle biogenesis [46]. In neuroblastoma, NEAT1_1 and NEAT1_2 seem to have opposing functions in terms of tumorigenesis, in which NEAT1_1 acts as an oncogene and NEAT1_2 acts as a tumor suppressor [47]. Additionally, Yan et al. found that NEAT1_2 remains in the nucleus, but NEAT1_1 is released from the nucleus into the cytoplasm of acute myeloid leukemia (AML) cells [48]. Therefore, considering the differential role of the two NEAT1 isoforms in the platinum resistance of OC, we downregulated the expression of NEAT1 and NEAT1_2 in OC cells through siRNA. Our results demonstrated that NEAT1 silencing suppressed platinum resistance in OC cells, whereas NEAT1_2 silencing had no effect on platinum resistance, and platinum resistance could be rescued by overexpressing NEAT1_1, suggesting that NEAT1_1 downregulation might attenuate platinum resistance in OC cells. Regarding the mechanism, it is known that NEAT1 is an indispensable component of nuclear paraspeckles, but only the NEAT1 long isoform NEAT1_2 is essential for the assembly of paraspeckles [49]. Based on the immunofluorescence of the paraspeckle proteins PSPC1 and SFPQ, we found that CSTF3 knockdown did not affect the aggregation of proteins, and silencing NEAT1 or NEAT1_2 led to reduced protein aggregation for phase separation. These results indicated that the downregulation of NEAT1_2 caused the disintegration of paraspeckles, but it had no effect on the platinum resistance of OC cells. Thus, we further propose that the progression of platinum resistance in OC cells was independent of the formation and function of paraspeckles.

How CSTF3-mediated APA processing of NEAT1 participates in the platinum resistance of OC cells. On the other hand, we observed that the PI3K/AKT pathway was significantly enriched for differentially expressed genes after downregulating the expression of CSTF3, NEAT1 or NEAT1_2 in OC cells. In previous studies, the PI3K/AKT pathway was shown to be a classical oncogenic signaling pathway that is involved in the occurrence, development and chemotherapy resistance of various tumors, and downregulated expression of NEAT1 inhibited the PI3K/AKT/mTOR signaling pathway in many cancer cells [50–52]. In our study, CSTF3 or NEAT1 knockdown resulted in decreased protein levels of p-PI3K, p-AKT and p-mTOR, whereas there were no significant changes in the protein levels of PI3K, AKT, mTOR, p-PI3K, p-AKT and p-mTOR following only knockdown of NEAT1_2. These results primarily confirmed that CSTF3 activated the PI3K/AKT/mTOR signaling pathway by regulating the APA processing of NEAT1, and the activated PI3K/AKT/mTOR pathway further promoted the platinum resistance of OC.

However, our study still has several limitations. Because the 5’ region of NEAT1 is common to the two isoforms, it is difficult to reduce the expression of NEAT1_1 individually. Thus, the NEAT1 5’ primers detected both NEAT1_1 and NEAT1_2, while the 3’ primers detected only NEAT1_2. The long isoform NEAT1_2 is 23 kb in length, so constructing the overexpression plasmid is difficult. In addition, we only focused on the APA regulation of NEAT1, and other target genes may also affect the progression of platinum resistance in OC cells. Therefore, in future studies, we will investigate the functions of more CSTF3 targets in the platinum resistance of OC.

**Fig. 8:**
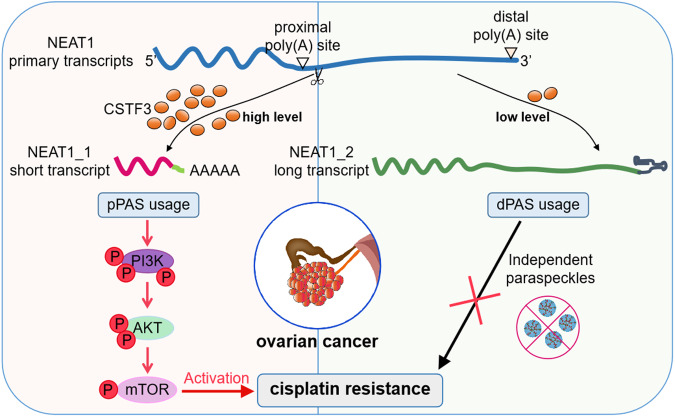
**Diagram of the regulatory mechanism and function underlying CSTF3 mediating the platinum resistance of OC cells**.

In conclusion, we revealed that the APA regulator CSTF3 played an important role in the platinum resistance of OC by affecting the APA processing of NEAT1. Increased CSTF3 expression tended to generate the short isoform NEAT1_1 by binding to the NEAT1 PAS and regulating APA. CSTF3 knockdown attenuated the platinum resistance of OC cells, and overexpression of NEAT1_1 recovered the resistance to platinum. Moreover, our study suggested that CSTF3 and NEAT1_1 exert their function for platinum resistance in OC cells via activation of the PI3K/AKT/mTOR pathway. A schematic model was shown in Fig. 8. Consequently, targeting CSTF3 and NEAT1_1 might be a promising therapeutic strategy for platinum-resistant OC and improve the prognosis of patients.

## Materials and methods

### Analysis of public datasets

The dataset of chemoresistant OC was downloaded from the GEO database (GSE154600). This dataset contained 2 platinum-resistant, 2 platinum-sensitive and 1 refractory tissue from 10X Genomics single-cell RNA sequencing (scRNA-seq) of high-grade serous ovarian carcinoma (HGSOC). We selected platinum-resistant and platinum-sensitive cells to perform transcriptome expression and APA analysis. The scRNA-seq data were analyzed by the R package Seurat3.0. Then, the t-SNE and UMAP dimensionality reductions were performed. Following a previous report [28], cell type annotation was performed using SingleR, and cancerous epithelial cells were captured to measure the length of transcripts and the expression of genes. The different lengths and differentially expressed transcripts between platinum-resistant and platinum-sensitive samples were assessed using Dapars2 and DESeq2, respectively.

### Cell culture and construction of platinum-resistance cell

A2780, OVCAR3 and HEK 293 T (293 T) cells were purchased from the National Infrastructure of Cell Line Resource (Beijing, China). Cell lines were authenticated by STR profiling. The platinum-resistant OC cells A2780-DDP and OVCAR3-DDP were induced by culturing with increasing concentrations of cisplatin (DDP) from wild type (WT) A2780 and OVCAR3 for a long time. A2780, OVCAR3 and corresponding platinum-resistance cells were cultured in RPMI 1640 (Gibco) medium, and 293 T cells were maintained in DMEM (Gibco) supplemented with 10% FBS (Biological Industries) and 1% penicillin/streptomycin (Sigma). And the platinum-resistance cells medium additionally contained low concentration DDP to maintain the drug-resistant phenotype. All cells with fresh medium were cultured at 37 °C in a humidified incubator within a 5% CO_2_ environment.

### Plasmid transfection and lentiviral infection

To produce lentivirus with shRNA, complementary sense and antisense oligonucleotides targeting CSTF3 were synthesized by Tsing Ke Biotechnology (Chongqing, China), annealed and cloned and inserted into the pLKO.1 vector (Addgene). NEAT1 siRNA and NEAT1_2 siRNA were synthesized by GenePharma (Shanghai, China). The shRNA and siRNA sequences were shown in Table S1. To knockdown CSTF3, 293 T cells were transfected with shRNA plasmids, psPAX2 plasmid and pMD 2. G plasmid by jetPRIME reagent (Polyplus Transfection), then the collected lentiviral production was used to infect cells. The CSTF3 and NEAT1_1 overexpression plasmids were purchased from WZ Biosciences (Shandong, China) and transfected using jetPRIME reagent. All of the corresponding empty plasmids acted as negative controls.

### Protein isolation and western blot

The total proteins of cells were obtained within RIP buffer (Beyotime, China) containing 1% phenylmethyl sulfonyl fluoride (PMSF) and quantified by a BCA detection kit (Solarbio, China). Equal amounts of protein were separated by 10% sodium dodecyl sulfate‒polyacrylamide gel electrophoresis (SDS‒PAGE) and transferred to polyvinylidene fluoride (PVDF) membranes (Millipore, USA). The membrane was blocked in 5% skim milk for 1 h and incubated with primary antibodies overnight at 4 °C. The primary antibodies were as follows: CSTF3 (Bethyl, A301-096A), PI3K (Abcam, ab151549), AKT (Proteintech, 60203-2-Ig), mTOR (CST, 2983 T), phospho-PI3K (CST, 4228 S), phospho-AKT (CST, 4060 S), phospho-mTOR (CST, 5536 S), FLAG-tag (MBL, M185-3L, PM020), and GAPDH (Proteintech, 60004-1-Ig). The next day, the membrane was incubated with the corresponding secondary antibody at room temperature for 1 h. The positive band was then detected by chemiluminescence. Densitometry was used to quantify the intensity of each band relative to that of GAPDH with ImageJ software.

### RNA extraction and RT-qPCR

TRIzol reagent (Invitrogen) was used to extract total RNA from OC cells. For the RT-qPCR assay, RNA was reverse-transcribed using a cDNA Synthesis Kit (Vazyme, China). Then, the cDNA quantity was detected with Universal SYBR qPCR Master Mix (Vazyme, Q711-02), and the reaction was performed on a 7500 Real Time PCR system (ABI) according to the manufacturer’s instructions. The primers were synthesized at Tsing Ke (Chongqing, China), and the sequences were shown in Table S2. All reactions were performed in triplicate. Finally, the relative expression of the target gene was calculated using the 2^−ΔΔCt^ method, with GAPDH as the internal reference.

### Immunohistochemistry (IHC) and HE staining

The OC xenograft specimens were fixed in 4% paraformaldehyde, embedded in paraffin, and cut into 4 μm thick sections. The sections were deparaffinized and rehydrated, heated in 100 °C citric buffer (pH 6.0) for 15 min to recover the antigen, and blocked with 5% bovine serum albumin (BSA) for 1 h. Then, they were incubated with anti-Ki-67 antibody (Servicebio, GB111141-100) and anti-Caspase-3 antibody (Servicebio, GB115600-100) at 4 °C overnight. The negative control group used phosphate-buffered saline (PBS) as the primary antibody. The next day, after incubation with HRP anti-rabbit IgG for 30 min at room temperature, the sections were stained with diaminobenzidine (DAB) and hematoxylin and mounted with resinene. The xenograft sections were also stained with hematoxylin and eosin (HE), the immunoreactivity was observed and photographed under a microscope.

### Immunofluorescence staining

Cells were placed in 24-well plates, fixed with 4% paraformaldehyde for 15 min, permeabilized with 0.2% Triton X-100 for 10 min, and then blocked with 1% bovine serum albumin (BSA) for 1 h at room temperature. Primary antibodies against CSTF3 (Proteintech, 24290-1-AP), SFPQ (Abcam, ab11825) and PSPC1 (Santa Cruz, sc-374181) were added and incubated overnight at 4 °C. After washing 3 times with PBS, the secondary antibodies Alex Fluor 647 Goat Anti-Mouse IgG(H + L) (Abcam, ab150115) and Alex Fluor 488 Goat Anti-Mouse IgG(H + L) (Abcam, ab150077) were added and cultured in the dark for 1 h at room temperature. DAPI (Abcam, ab104139) staining was performed for 5 min to visualize the cell nucleus. Finally, the cells were observed and photographed under a fluorescence microscope.

### Cell Counting Kit-8 (CCK-8) assay

The CCK-8 assay was used to measure proliferation of transfected cells. 2 × 10^3^ cells were placed in 96-well plates and cultured with 100 μL growth medium containing 10% FBS. After 0, 24, 48, 72, and 96 h of incubation, 10 μL of CCK-8 solution (Beyotime, China) was added to each well and incubated at 37 °C for 2 h. The absorbance value (optical density, OD) at 450 nm was measured on a microplate spectrophotometer (Bio-Rad, USA).

### Cell viability assay

The cell viability assay was used to measure drug resistance. The transfected cells were placed in 96-well plates at a density of 1 × 10^4^ cells per well and cultured in 100 μL growth medium containing 10% FBS overnight. The next day, the cells were incubated with gradient concentrations of cisplatin (DDP, MCE) for 48 h. Then, 10 μL of CCK-8 solution (Beyotime, China) was added to each well and incubated at 37 °C for 2 h. The absorbance value (optical density, OD) at 450 nm was measured on a microplate spectrophotometer (Bio-Rad, USA). Data were obtained from three parallel experiments. The IC50 of DDP, based on the OD value, was calculated using Prism 6 (GraphPad, USA) software.

### Colony formation assay

For the colony formation assay, approximately 2 × 10^3^ cells were seeded in 6-well plates overnight, followed by treatment with or without DDP (IC50) for 6 h. Then, the DDP medium was removed, and the complete medium was replaced for 2 weeks. The cells were fixed with 4% paraformaldehyde and stained with 0.5% crystal violet. Finally, the colonies were washed with PBS and recorded on a light board. The results were quantified by ImageJ software to analyze cell proliferation and drug resistance.

### Cell apoptosis assay

Cell apoptosis was detected using an Annexin V-FITC/PI Apoptosis Detection Kit (DOJINDO, AD10). Transfected cells were treated with DDP (2 μM) for 24 h to induce apoptosis, and cells were harvested and washed twice with cold PBS. Then, the cells were resuspended in 500 μL of binding buffer, stained with 5 μL of Annexin V/FITC and 5 μL of PI and incubated for 15 min at room temperature in the dark. Immediately, the stained cells were detected by flow cytometry (BD Biosciences, USA), and the apoptosis ratio was analyzed via FlowJo software.

### In vivo tumorigenicity and metastasis models

Four-week-old female nude mice were purchased from Tenxin Co., Ltd. (Chongqing, China), and all mice were randomly divided into NC and knockdown groups. For tumor xenograft formation, the corresponding 5 × 10^6^ transfected cells were subcutaneously injected into mice with 50% matrigel (BD Biosciences) in 100 μL of PBS. One week later, the tumor was formed, and the mice were randomly divided into control and treatment groups with five mice in each group and then received saline or 4 mg/kg DDP through intraperitoneal injection once every 6 days. The length and width of the tumors were monitored every 3 days, and the volume was calculated using the formula: tumor size (mm^3^) = smallest diameter^2^ × largest diameter/2. After 22 days, the mice were euthanized, and tumors were collected, weighed, and analyzed. All of the mouse experiments in this study were approved by the Institutional Animal Care and Use Committee of Chongqing Medical University (approval number IACUC-CQMU-2023-0153).

### RNA-seq

Total RNA was isolated from transfected cells with silenced and NC cells using TRIzol Reagent (Invitrogen) and sent to Majorbio (Shanghai, China) for RNA-seq analysis according to the standard Illumina protocol. The products were purified by the AMPure XP system, and the library quality was evaluated on the Agilent Bioanalyzer 2100 system. After generating clusters using the TruSeq PE Cluster Kit v3-cBot-HS, the libraries were sequenced on the Illumina HiSeq 2500 platform. Data analysis was conducted in the R programming environment. Analysis of differential expression was performed using the DESeq2 package, and the cutoffs for significantly differentially expressed genes were adjusted *p*-value < 0.05 and log2-fold change > 2. Then, the differentially expressed genes (DEGs) were subjected to Gene Ontology (GO) term and Kyoto Encyclopedia of Genes and Genomes (KEGG) pathway enrichment analyses using DAVID.

### PAS-seq

The efficiency of knockdown was authenticated, and mycoplasma contamination was tested in infected cells. PAS-seq was performed using the QuantSeq REV 3’ mRNA-Seq Library Prep Kit (Lexogen, 016.24) according to the manufacturer’s instructions. Briefly, 500 ng of total RNA was synthesized as first-strand cDNA with an oligodT primer containing an Illumina-compatible sequence in a PCR plate. The second-strand cDNA was synthesized by a random primer containing an Illumina-compatible linker sequence at its 5’ end, and the library was converted to dsDNA. The library was purified using magnetic beads and amplified to add the complete adapter sequences and unique indices for multiplexing. The finished library was purified from PCR components. Subsequently, the library was sent to Annoroad Gene Technology (Beijing, China) for detection and sequencing. The raw reads were trimmed and mapped to the human genome (Ensembl GRCh38.p5) using Bowtie2. For APA analysis, we assessed differential PAS reads using PolyA-miner to identify APA changes and transcripts with different lengths [53]. In addition, differential expression analysis was performed with DESeq2. The genes with length change and differential expression were identified by cutoff with adjusted *p-*value < 0.05.

### eCLIP-seq

Enhanced UV crosslinking immunoprecipitation (eCLIP) was performed following previously described methods with slight modifications [54]. Briefly, OVCAR3 cells transfected with the CSTF3-FLAG plasmid were UV-crosslinked at 150 mJ and 254 nm wavelengths with cold PBS. The cells were lysed with eCLIP lysis buffer and further treated with RNase I (1:2500, Invitrogen) and Turbo DNase (Invitrogen, AM2238) as described. After collecting 5% of the input, the lysate was incubated with 20 μg of anti-FLAG-tag (MBL, M185-3L) antibody overnight at 4 °C. Then, 140 μL of protein G beads (Invitrogen) was added and conjugated for 2 h at 4 °C. RNAs within beads were dephosphorylated at the 3′ end by T4 PNK (NEB, M0201L) and ligated to an RNA adaptor. Then, the immunoprecipitated RNA was separated using Nupage 4–12% Bis–Tris protein gels and transferred to nitrocellulose membranes. The matched size of protein‒RNA complexes and input protein were cut from the membrane. RNAs on the membrane were harvested by digesting proteins using proteinase K (Roche, 3115828001) and subsequently reverse transcribed using SuperScript III (Thermo Fisher, USA). Finally, cDNA libraries were amplified, purified, and sequenced by using an Illumina HiSeq 1000 with a paired-end 150 bp read length. eCLIP-seq was performed by Novogene Bioinformatics Technology (Tianjing, China). For eCLIP-seq analysis, the data were processed according to previous studies (https://github.com/YeoLab/eclip). The low-quality reads and adapter sequences were trimmed by cutadapt, and the repetitive reads were aligned by STAR. Next, the cleaned reads were mapped to the human genome (Ensembl GRCh38.p5) by STAR. Peak calling and downstream analysis were performed using clipper43 software. The peaks were filtered by adjusted *p*-value < 0.05 and log2-fold change > 8. The enrichment of CSTF3 binding sites was calculated by ChIPseeker39, and the motif enriched in CSTF3 binding sites was analyzed by HOMER37.

### Statistical analysis

All statistical analyses were conducted by GraphPad Prism 6. Data are presented as the mean ± SD of independent experiments. Data were analyzed using Fisher’s exact test, *the χ2* test, Student’s *t*-test, and one-way analysis of variance (ANOVA) as appropriate. The Kaplan–Meier method was used to analyze the cumulative survival time with an optimal cutoff value. The *p-*value < 0.05 was considered statistically significant for all statistical tests.

### Supplementary information

Supplementary information file in PDF

Full and uncropped western blots

STR profiling of cell lines

## Data Availability

The data were generated by the authors and available from JX (jingxu@hospital.cqmu.edu.cn) on request.
