# Supplementary material for: CSTF3 contributes to platinum resistance in ovarian cancer through alternative polyadenylation of lncRNA NEAT1 and generating the short isoform NEAT1_1
Source: Cell Death Dis. 2024 Jun 19;15(6):432. doi: 10.1038/s41419-024-06816-1 (PMC11187223; doi:10.1038/s41419-024-06816-1)
Supplement: Supplementary file 1 — Supplementary information file in PDF [file 41419_2024_6816_MOESM1_ESM.pdf]

# Supplementary information

## CSTF3 contributes to platinum resistance in ovarian cancer through alternative polyadenylation of lncRNA NEAT1 and generating the short isoform NEAT1_1

Xin Luo[1,4], Qinglv Wei[2,4], Xiaoyan Jiang[1,4], Ningxuan Chen[1], Xinzhao Zuo[1], Hongyan Zhao[3], Yujiao Liu[1], Xiaoyi Liu[1], Lingcui Xie[1], Yu Yang[1], Tao Liu[1✉], Ping Yi[1✉], Jing Xu[1✉]

[1]Department of Obstetrics and Gynecology, The Third Affiliated Hospital of Chongqing Medical University, Chongqing, China. [2]Chongqing Key Laboratory of Child Infection and Immunity, Children's Hospital of Chongqing Medical University, Chongqing, China. [3]School of Basic Medicine, Hubei University of Medicine, Shiyan, Hubei, China. [4]These authors contributed equally: Xin Luo, Qinglv Wei, Xiaoyan Jiang. ✉email: anti1988@163.com; yiping@cqmu.edu.cn; jingxu@hospital.cqmu.edu.cn

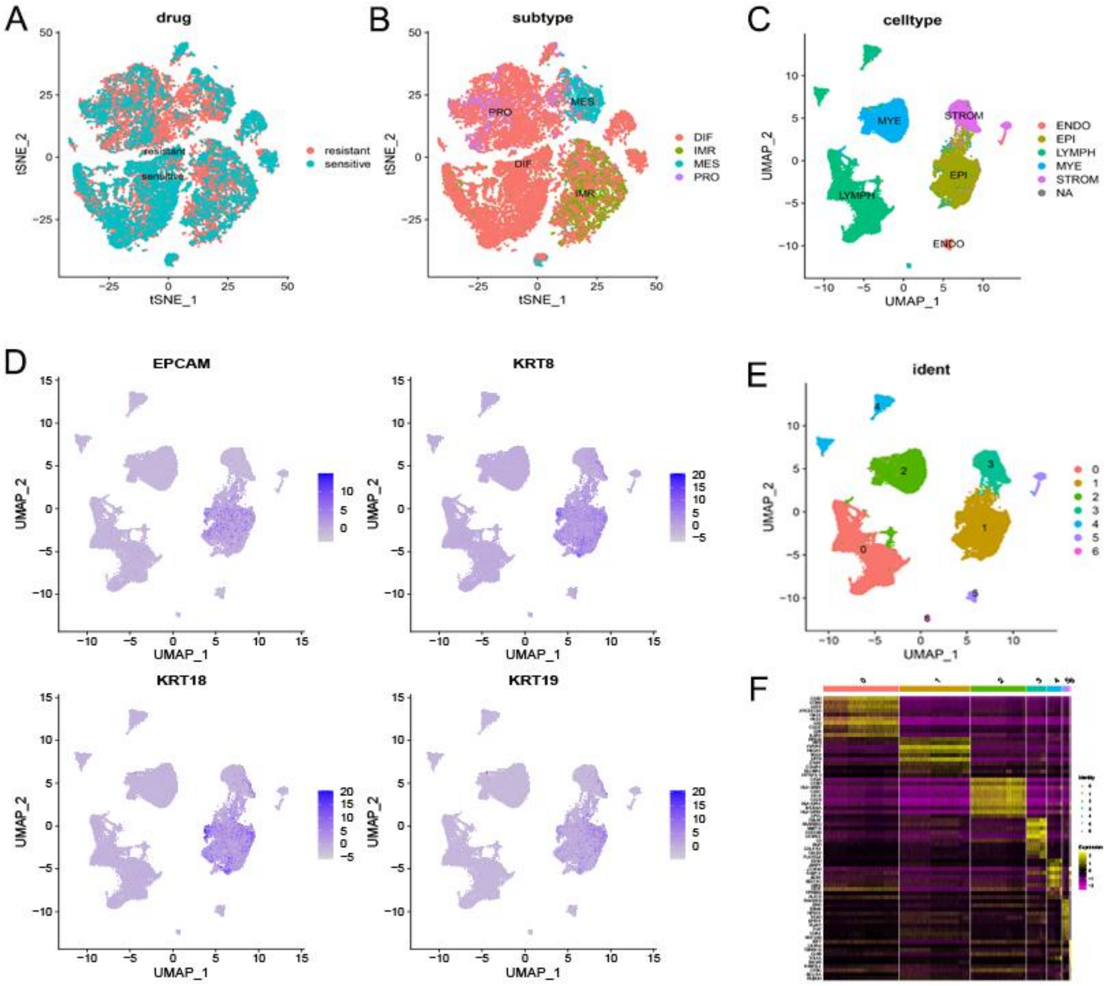


**Figure S1. scRNA-seq analysis with the platinum resistance and sensitivity of OC datasets.** Integrating all scRNA-seq data, dimension reduction by tSNE or UMAP aimed at drug treatment (A), subtype (B) and cell type (C). (D) The epithelial marker genes EPCAM, KRT8, KRT18, and KRT19 annotated epithelial cell (EPI) using UMAP. (E) Cells were clustered based on the expression of highly variable genes and visualized using UMAP, and heatmap of average ten specific genes show the features of each cluster (F).

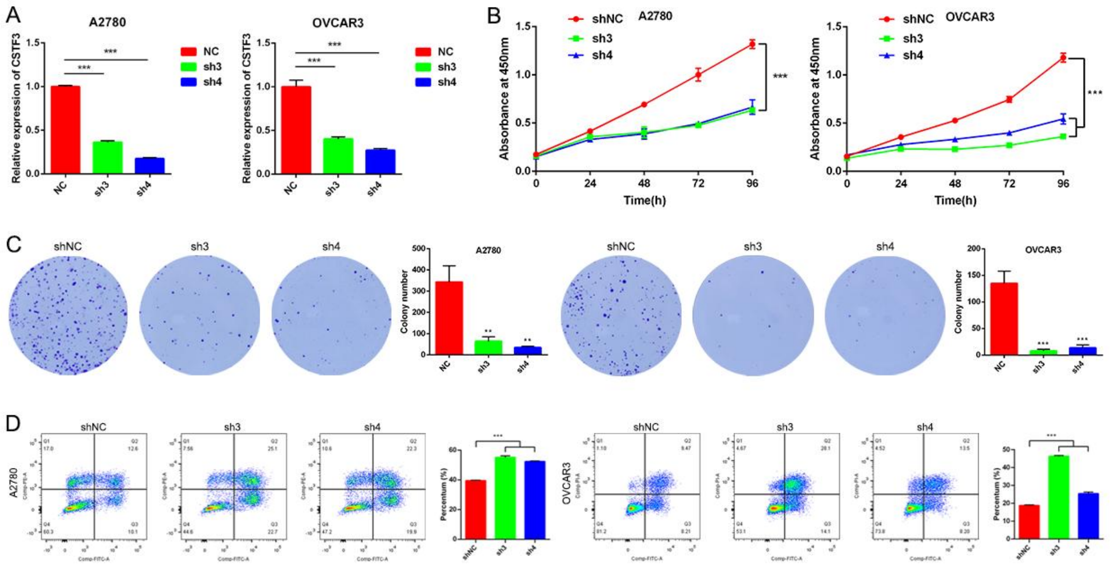


**Figure S2. CSTF3 knockdown suppressed OC cell proliferation and induced apoptosis.** A. CSTF3 mRNA was measured to confirm the knockdown efficiency in A2780 and OVCAR3 cells. CCK8 (B) and colony formation (C) assays were performed to determine the proliferation of OC cells after downregulation of CSTF3 expression. (D) Apoptosis was measured by flow cytometry. *$P < 0.05$, **$P < 0.01$, ***$P < 0.001$ vs. shNC. ns not significant.

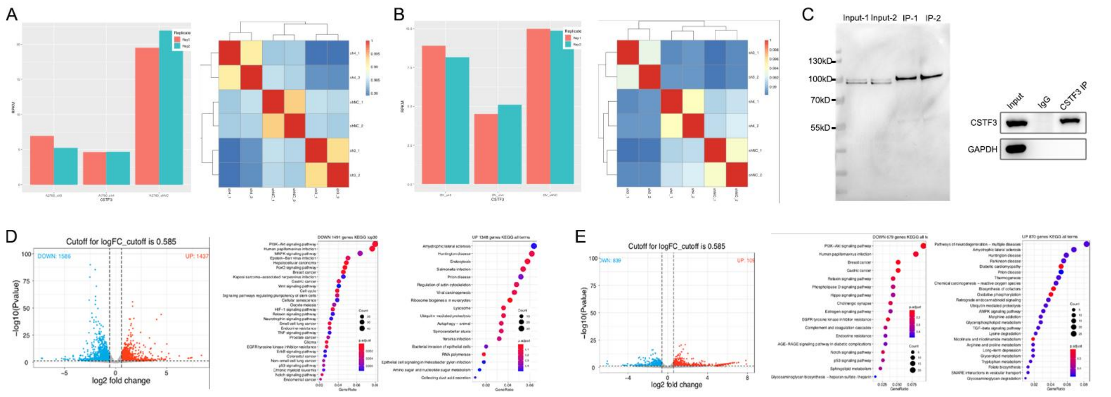


**Figure S3. Analysis of PAS-seq with CSTF3 knockdown and quality control of CSTF3 eCLIP-seq and eCLIP-qPCR.** (A) The knockdown efficiency of the CSTF3 gene and the correlation of knockdown and control samples in PAS-seq of A2780 cells. (B) The image of knockdown efficiency and heatmap of the samples' correlation in OVCAR3 cells. (C) Immunoprecipitation of CSTF3 eCLIP-seq and eCLIP-qPCR. (D, E) Analysis of differentially expressed genes as well as KEGG pathway enrichment with upregulated and downregulated genes for PAS-seq results of A2780 and OVCAR3 cells.

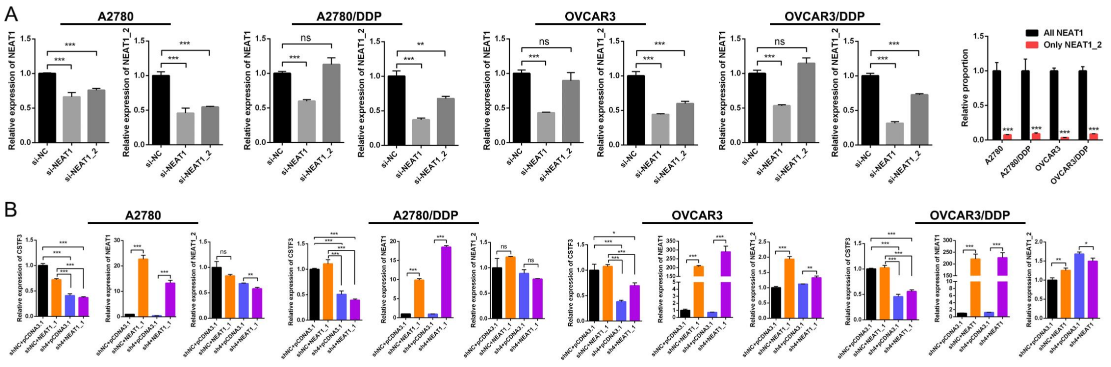


**Figure S4. Silencing NEAT1 and NEAT1_2 in A2780, OVCAR3 and corresponding platinum-resistant cells as well as overexpressing NEAT1_1 in A2780, OVCAR3 and platinum-resistant cells with CSTF3 knockdown.** (A) The silencing verification and expression examination of NEAT1 and NEAT1_2 in A2780, OVCAR3 and corresponding platinum-resistant cells by RT-qPCR. (B) The expression of CSTF3, NEAT1 and NEAT1_2 were detected when NEAT1_1 was overexpressed in A2780, OVCAR3 and platinum-resistant cells with CSTF3 knockdown. **P* < 0.05, ***P* < 0.01, ****P* < 0.001.

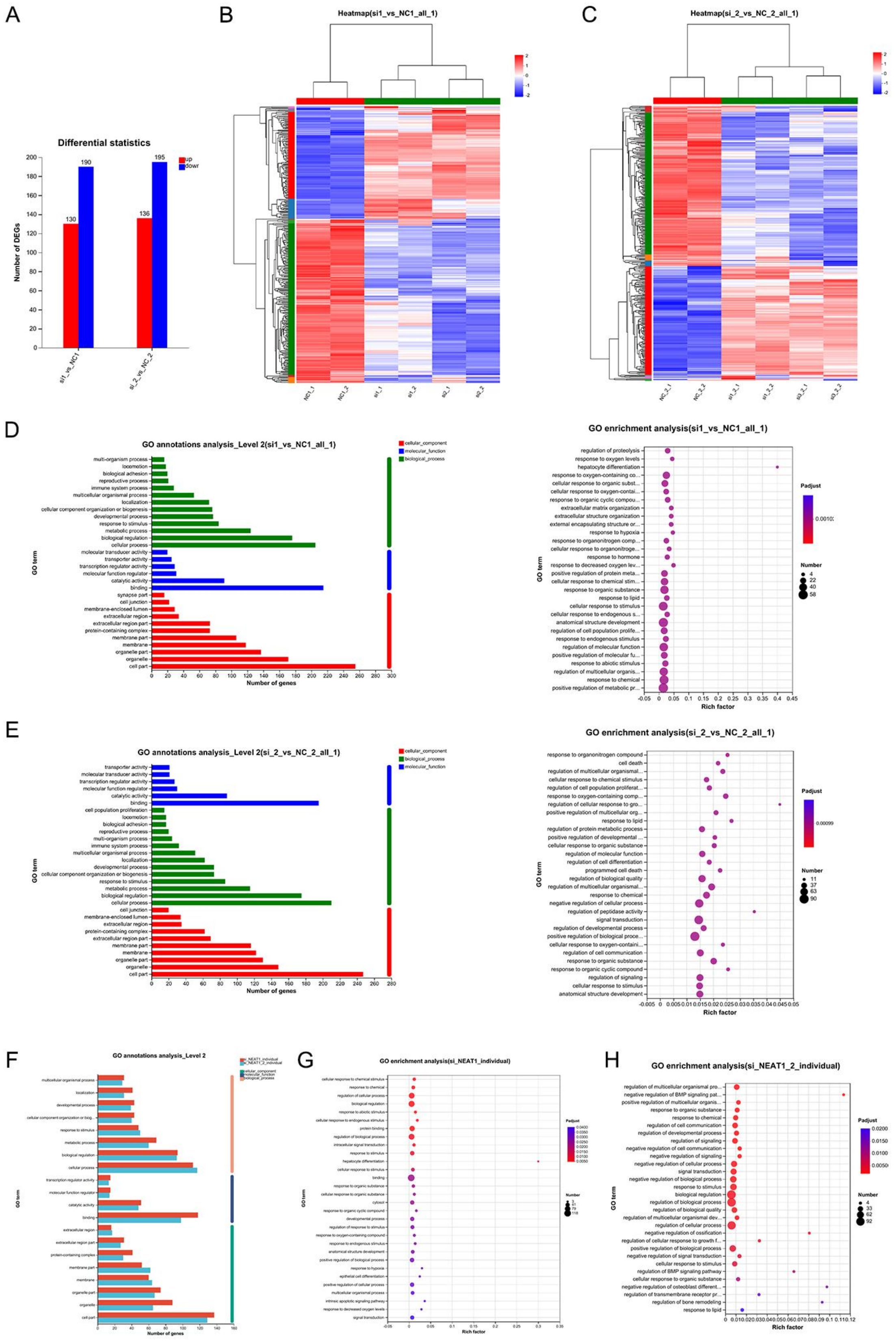


**Figure S5. Enrichment analysis of DEGs after silencing NEAT1 or NEAT1_2 in OVCAR3 cells.** (A) Histogram of DEGs after silencing NEAT1 and NEAT1_2 in OVCAR3 cells. (B, C) Heatmap of DEGs from OVCAR3 cells transfected with NEAT1 siRNA, NEAT1_2 siRNA and

control siRNA. (D) GO and KEGG enrichment analysis of DEGs for silencing NEAT1 in OVCAR3 cells. (E) GO and KEGG enrichment analysis of DEGs for silencing NEAT1_2 in OVCAR3 cells. (F) GO annotation analysis of independent DEGs with NEAT1 and NEAT1_2 silenced. (G) GO enrichment analysis of the individual DEGs list with NEAT1 silenced. (H) GO enrichment analysis of the individual DEGs list with NEAT1_2 silenced.

**Supplementary tables**

**Table S1. Sequences of oligos by interfering CSTF3, NEAT1 and NEAT1_2**

| Target names | | Sequences (5'–3') |
|---|---|---|
| CSTF3 shRNA-3 | Forward | CCGGGTGATGAAGCTGCTAATATATCTCGAGATATATT AGCAGCTTCATCACTTTTTG |
| | Reverse | AATTCAAAAAGTGATGAAGCTGCTAATATATCTCGAG ATATATTAGCAGCTTCATCAC |
| CSTF3 shRNA-4 | Forward | CCGGGCCCGATTTCTAGCATTTGAACTCGAGTTCAAAT GCTAGAAATCGGGCTTTTTG |
| | Reverse | AATTCAAAAAGCCCGATTTCTAGCATTTGAACTCGAGT TCAAATGCTAGAAATCGGGC |
| si-NC | Forward | UUCUCCGAACGUGUCACGUTT |
| | Reverse | ACGUGACACGUUCGGAGAATT |
| NEAT1 siRNA-1 | Forward | CAGGAGGCUACCAUUUAAATT |
| | Reverse | UUUAAAUGGUAGCCUCCUGTT |
| NEAT1 siRNA-2 | Forward | GCAGGUUGAAGGGAAUUCUTT |
| | Reverse | AGAAUUCCCUUCAACCUGCTT |
| NEAT1_2 siRNA-1 | Forward | CCAGGUCUGUCAAUAUUAATT |
| | Reverse | UUAAUAUUGACAGACCUGGTT |
| NEAT1_2 siRNA-2 | Forward | GGCCUCAUAUAAGUGUAAUTT |
| | Reverse | AUUACACUUAUAUGAGGCCTT |

**Table S2. Sequences of primers used in this study**

| **Gene** | **Primer** | **Sequence** (5'–3') |
|---|---|---|
| Primers for RT-qPCR | | |
| CSTF3 | Forward | CTGAGTATGTCCCAGAGAAGGT |
| | Reverse | TGCTCCAAGCATCAAGGTCAT |
| NEAT1 Distal | Forward | GGTGGGACTGTTCTGTCCTTG |
| | Reverse | AACAGGCCCAGGTGAGTAGA |
| NEAT1 Proximal | Forward | TGGGCGAGGTGCCTTTACTA |
| | Reverse | CCCAGAAGACAGAAAGATCCCA |
| GAPDH | Forward | GTCAAGGCTGAGAACGGGAA |
| | Reverse | AAATGAGCCCCAGCCTTCTC |
| Primers for eCLIP-qPCR | | |
| NEAT1-1 | Forward | TCGACCCCTATCACATTGCC |
| | Reverse | ACTTGGAGCTAGCAAATCTAGACC |
| NEAT1-2 | Forward | GGGATCTTTCTGTCTTCTGGGTT |
| | Reverse | ATACCCGAGACTACTTCCCCA |
| NEAT1-3 | Forward | GGTGGGGAGTACTTTGCCATA |
| | Reverse | CAAATCCCAGGCACATTCCAG |
