# Supplementary material for: CSTF3 contributes to platinum resistance in ovarian cancer through alternative polyadenylation of lncRNA NEAT1 and generating the short isoform NEAT1_1
Source: Cell Death Dis. 2024 Jun 19;15(6):432. doi: 10.1038/s41419-024-06816-1 (PMC11187223; doi:10.1038/s41419-024-06816-1)

Fig. 2B

CSTF3(77kD)

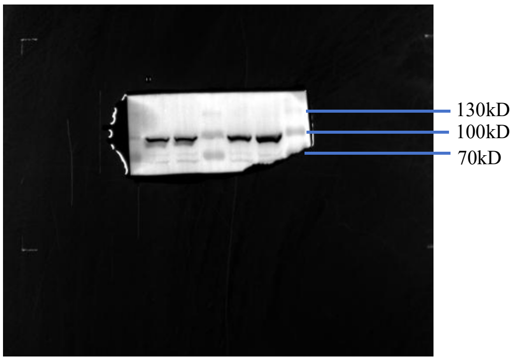

GAPDH(37kD)

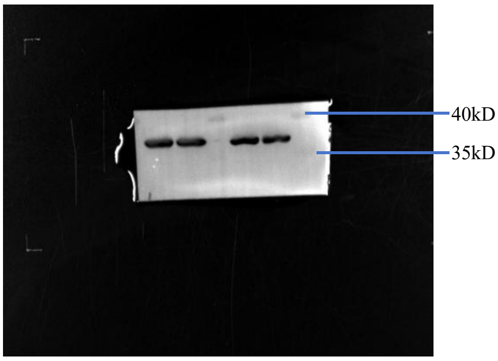

Fig. 2D

CSTF3(77kD)

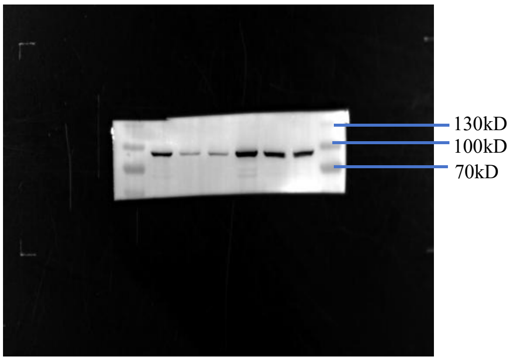

GAPDH(37kD)

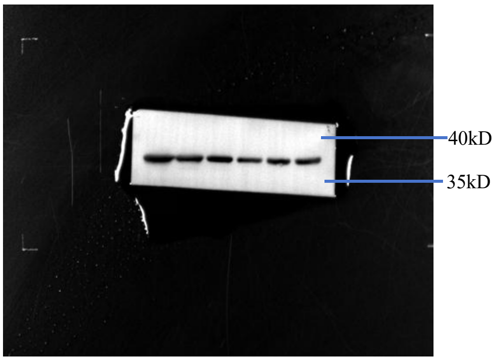

Fig. 2G

CSTF3(77kD)

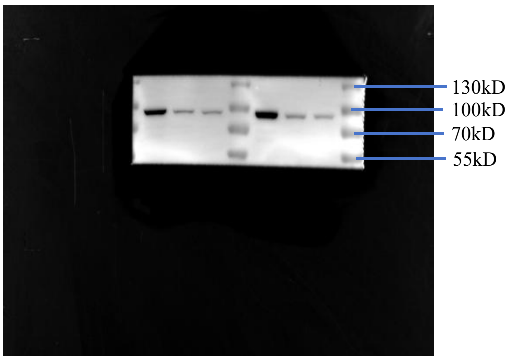

GAPDH(37kD)

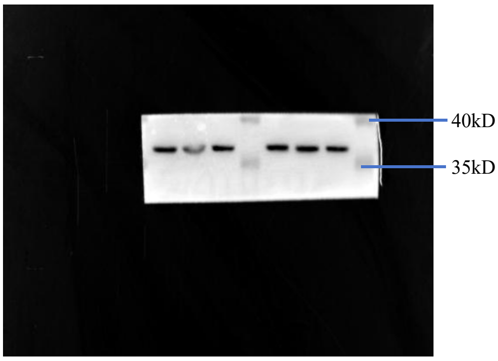

Fig. 2J

CSTF3(77kD)

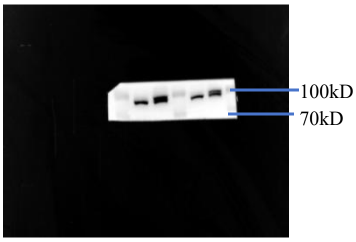

GAPDH(37kD)

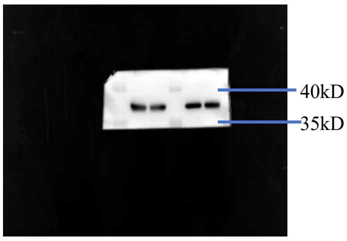

FLAG(77kD)

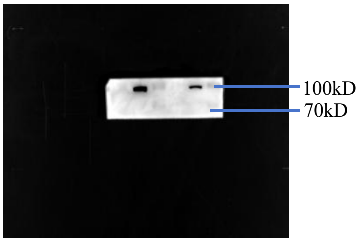

Fig. 7I

mTOR(289kD)

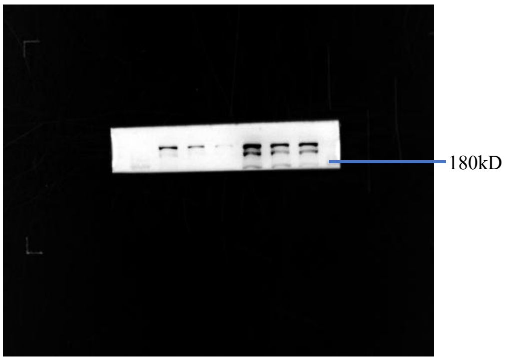

p-mTOR(289kD)

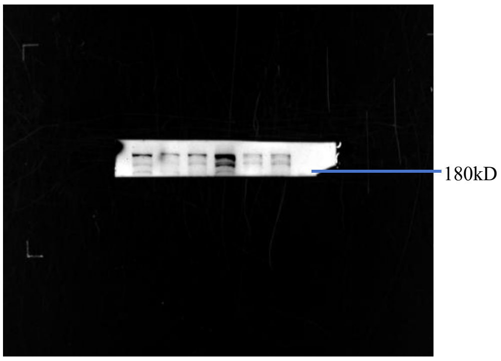

PI3K(85kD)

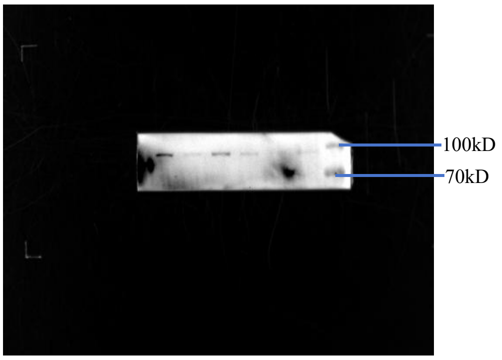

p-PI3K(85kD)

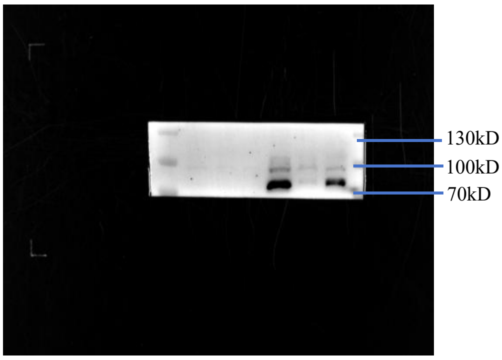

AKT(60kD)

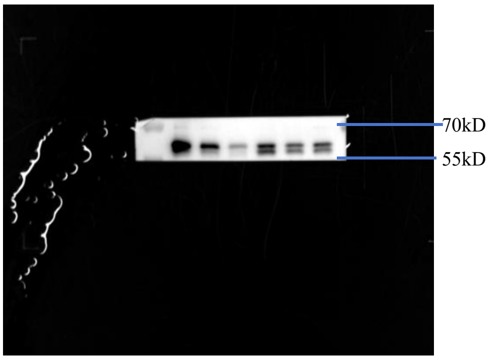

p-AKT(60kD)

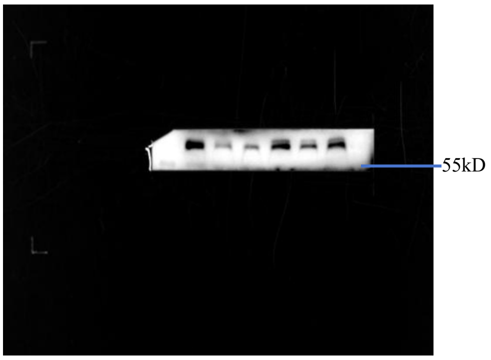

CSTF3(77kD)

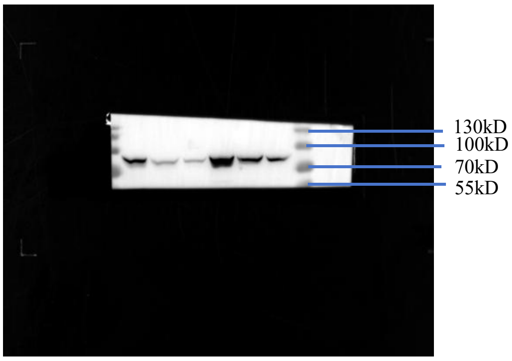

GAPDH(37kD)

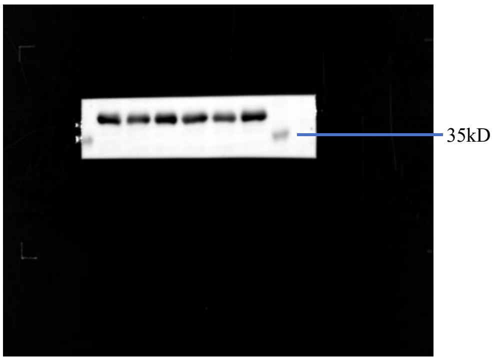

Fig. 7J

A2780 cell

mTOR(289kD)

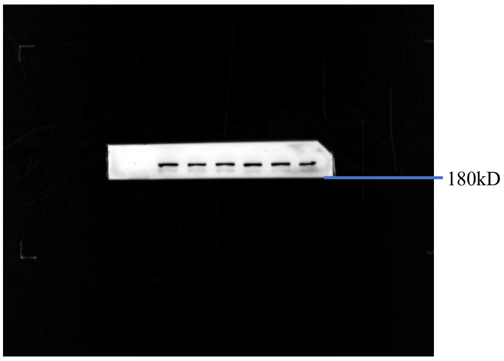

p-mTOR(289kD)

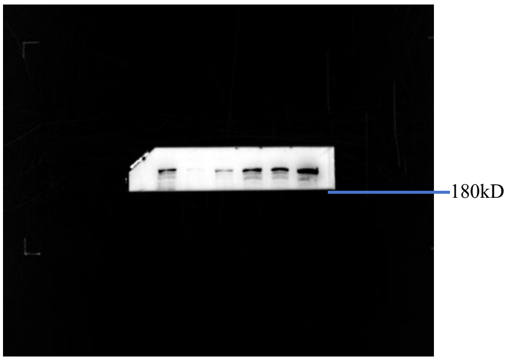

PI3K(85kD)

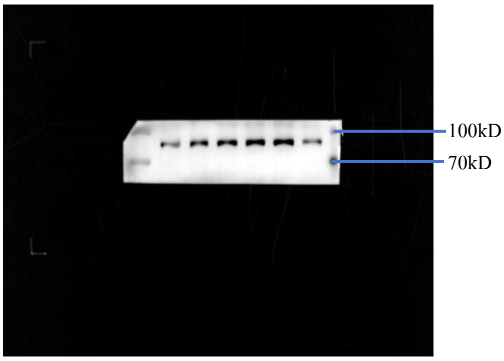

p-PI3K(85kD)

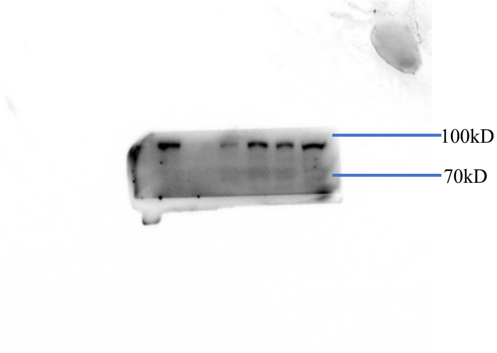

AKT(60kD)

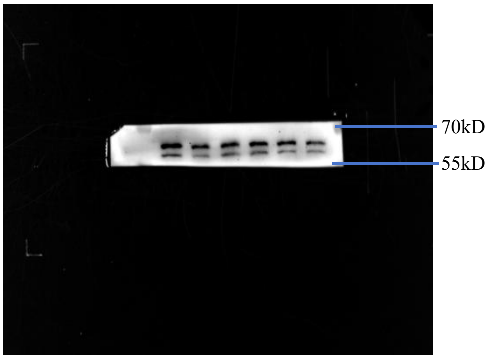

p-AKT(60kD)

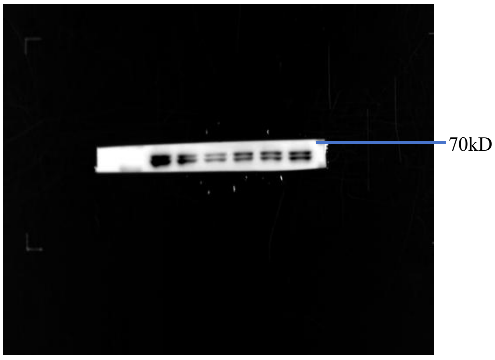

GAPDH(37kD)

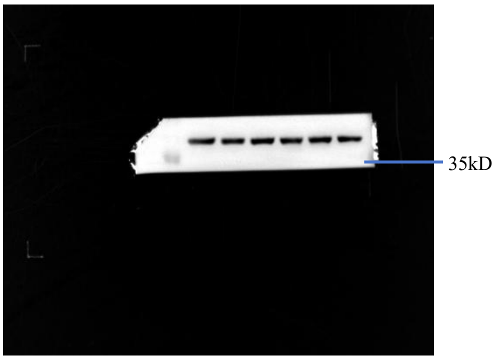

GAPDH-p(37kD)

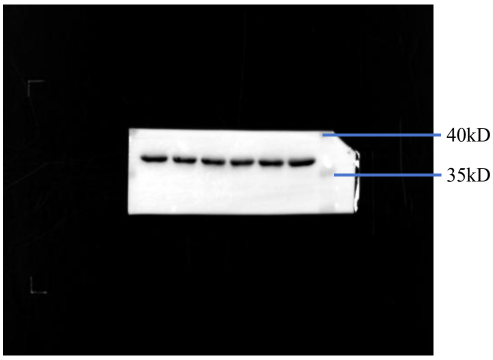

OVCAR3 cell

mTOR(289kD)

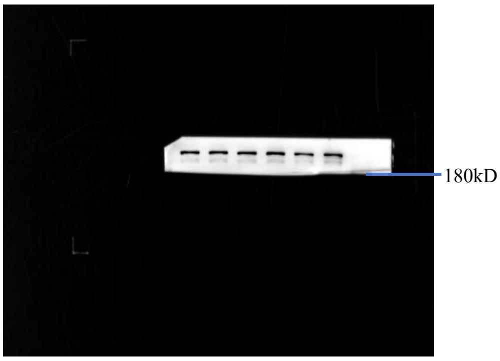

p-mTOR(289kD)

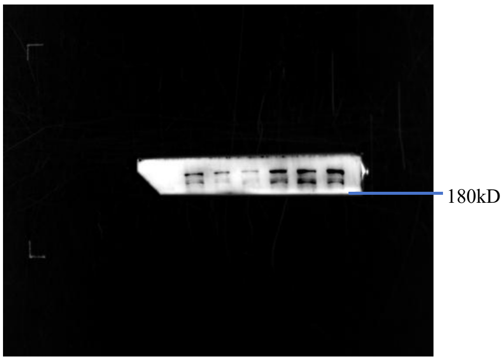

PI3K(85kD)

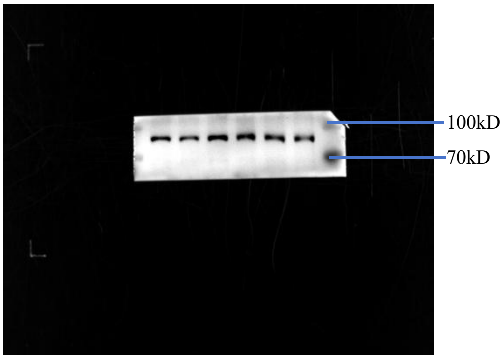

p-PI3K(85kD)

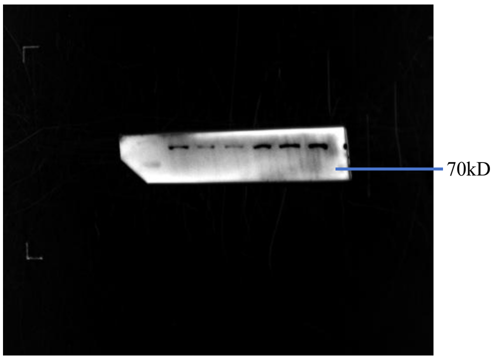

AKT(60kD)

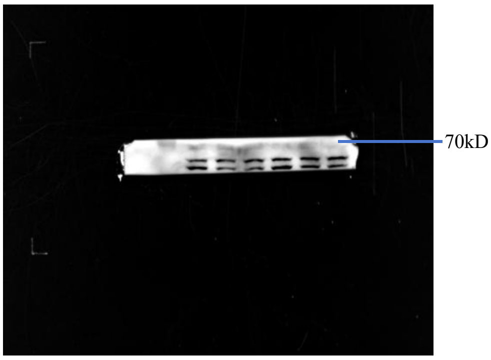

p-AKT(60kD)

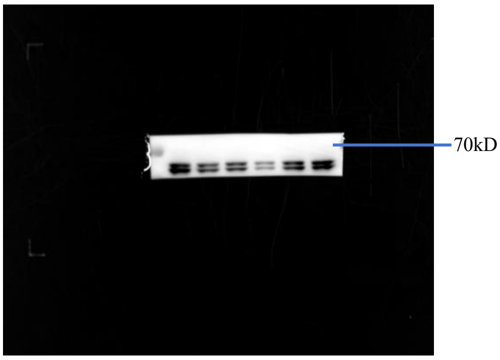

GAPDH(37kD)

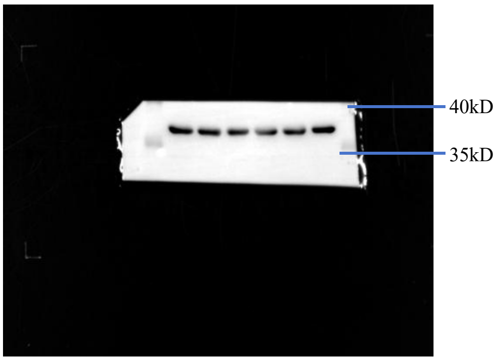

GAPDH-p(37kD)

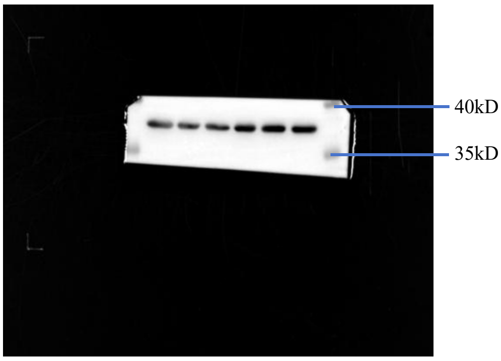

Sup Fig3. C

CSTF3-FLAG

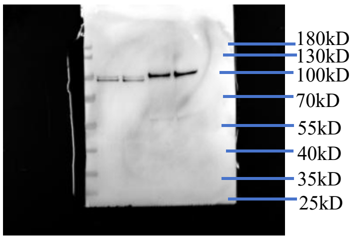

CSTF3

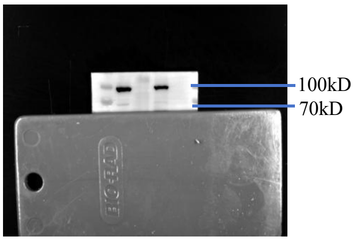

GAPDH(37kD)

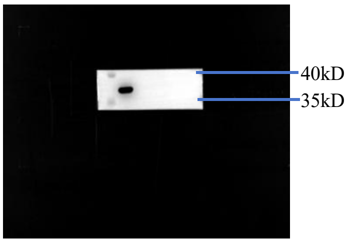

Supplement: Supplementary file 2 — Full and uncropped western blots [file 41419_2024_6816_MOESM2_ESM.pdf]
