# Supplementary material for: CSTF3 contributes to platinum resistance in ovarian cancer through alternative polyadenylation of lncRNA NEAT1 and generating the short isoform NEAT1_1
Source: Cell Death Dis. 2024 Jun 19;15(6):432. doi: 10.1038/s41419-024-06816-1 (PMC11187223; doi:10.1038/s41419-024-06816-1)

Applied Biosystems
GeneMapper ID v3.2

20230626

| Sample File | Sample Name | Panel | OS | SQ |
|---|---|---|---|---|
| 32_H04_Cellidentification-1---0627.fsa | A2780 | 21Plex_STR_Panel_v1.2 | | |

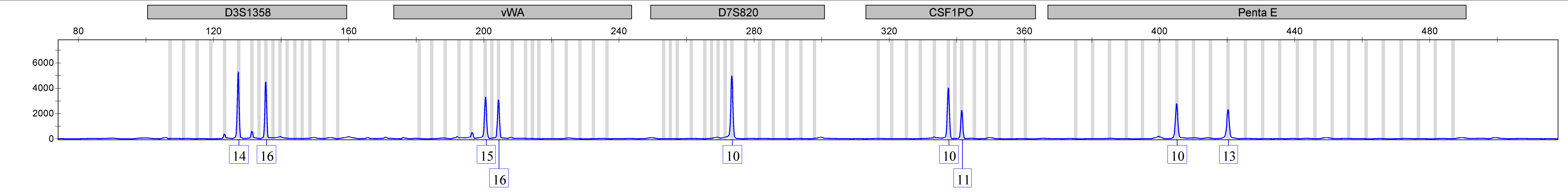


| | | | | |
|---|---|---|---|---|
| 32_H04_Cellidentification-1---0627.fsa | A2780 | 21Plex_STR_Panel_v1.2 | | |

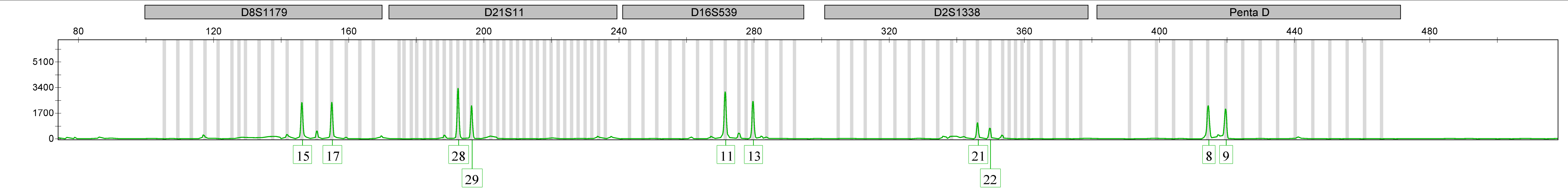


| | | | | |
|---|---|---|---|---|
| 32_H04_Cellidentification-1---0627.fsa | A2780 | 21Plex_STR_Panel_v1.2 | | |

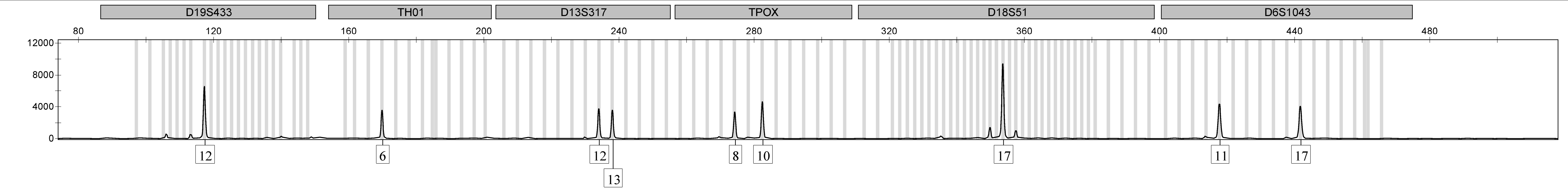


| | | | | |
|---|---|---|---|---|
| 32_H04_Cellidentification-1---0627.fsa | A2780 | 21Plex_STR_Panel_v1.2 | | |

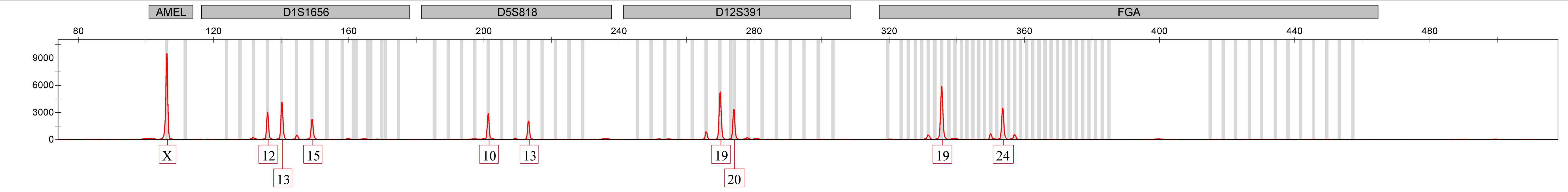

| Sample File | Sample Name | Panel | OS | SQ |
|---|---|---|---|---|
| 29_E04_CellLineAuthentication-0515.fsa | OVCAR-3 | STR Profile 1-human | | |

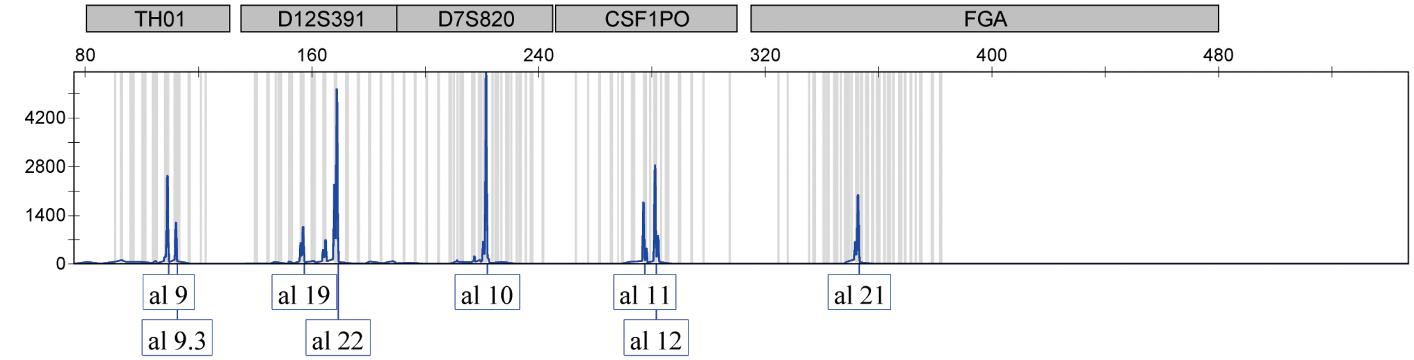


| Sample File | Sample Name | Panel | OS | SQ |
|---|---|---|---|---|
| 29_E04_CellLineAuthentication-0515.fsa | OVCAR-3 | STR Profile 1-human | | |

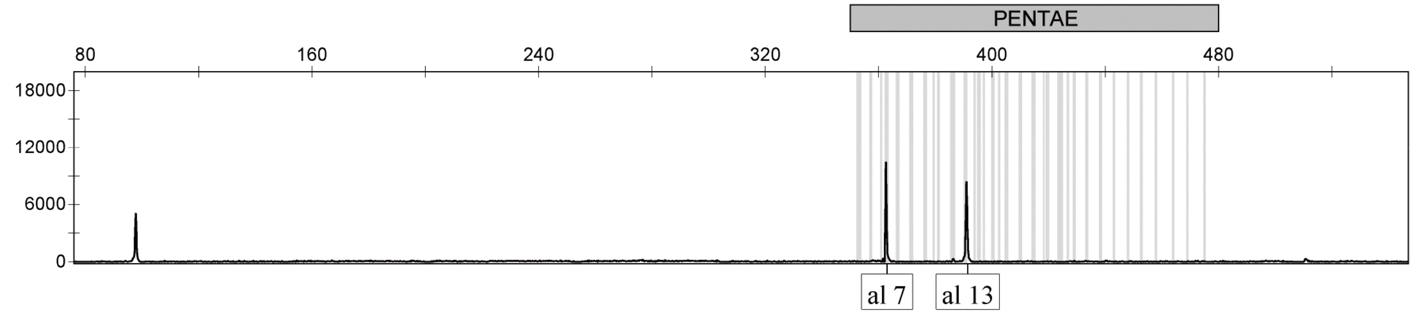


| Sample File | Sample Name | Panel | OS | SQ |
|---|---|---|---|---|
| 62_F08_CellLineAuthentication-0515.fsa | OVCAR-3 | STR Profile 2-human | | |

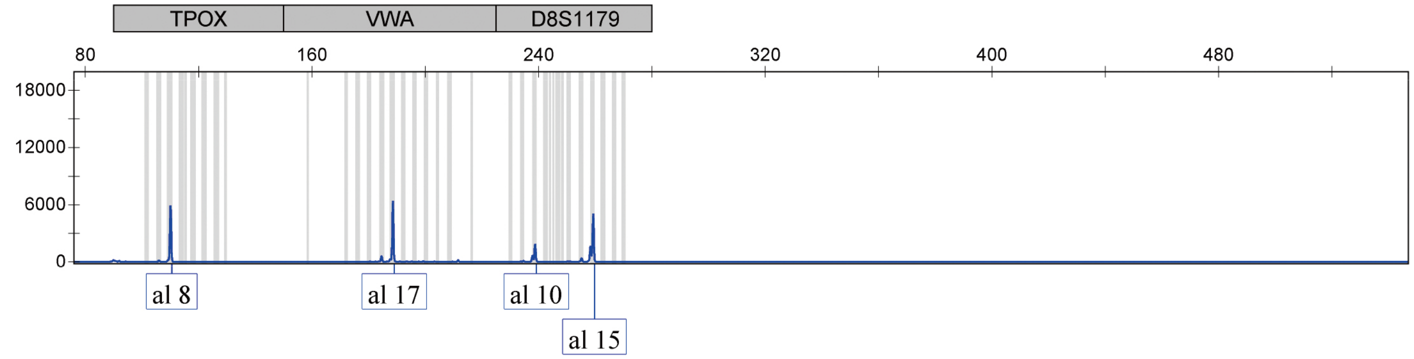

Applied Biosystems

GeneMapper 4.0

CELL-13

| Sample File | Sample Name | Panel | OS | SQ |
|---|---|---|---|---|
| 62_F08_CellLineAuthentication-0515.fsa | OVCAR-3 | STR Profile 2-human | | |

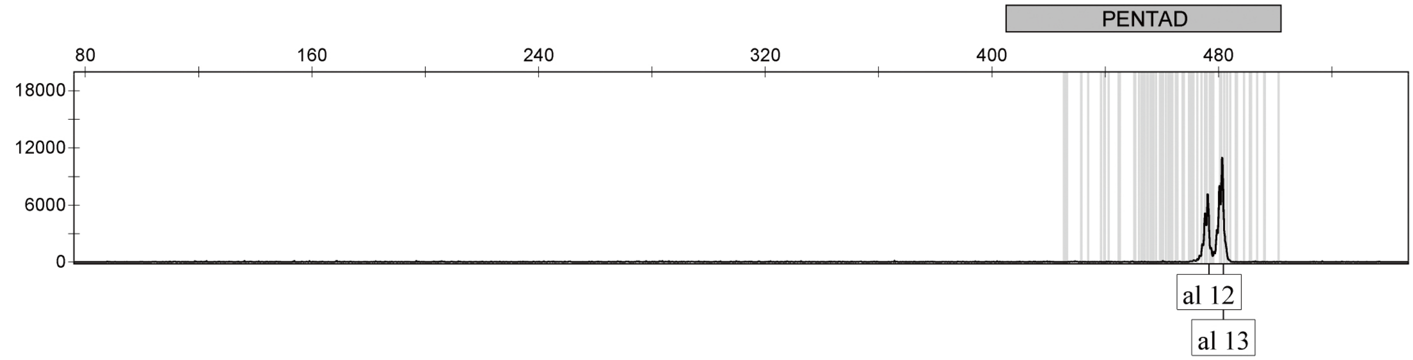


| | | | | |
|---|---|---|---|---|
| 92_D12_CellLineAuthentication-0515.fsa | OVCAR-3 | STR Profile 3-human | | |

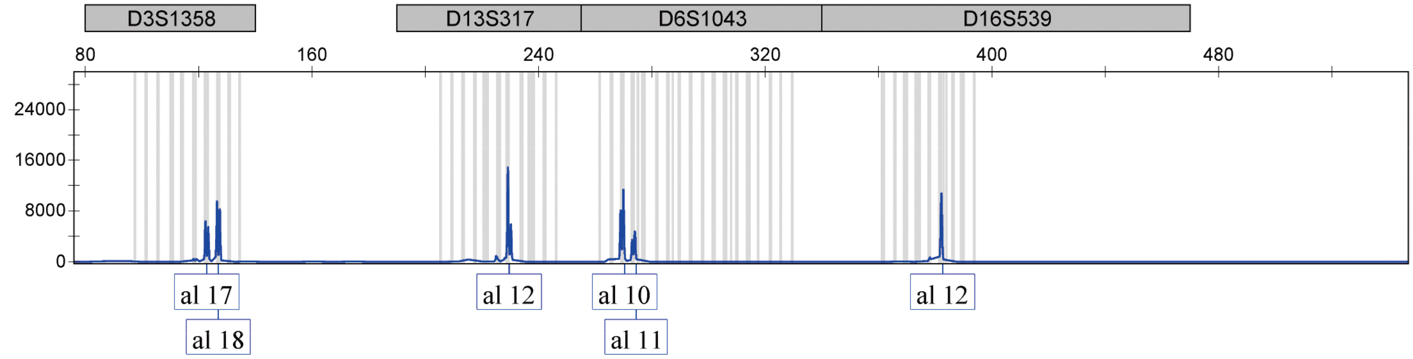


| | | | | |
|---|---|---|---|---|
| 92_D12_CellLineAuthentication-0515.fsa | OVCAR-3 | STR Profile 3-human | | |

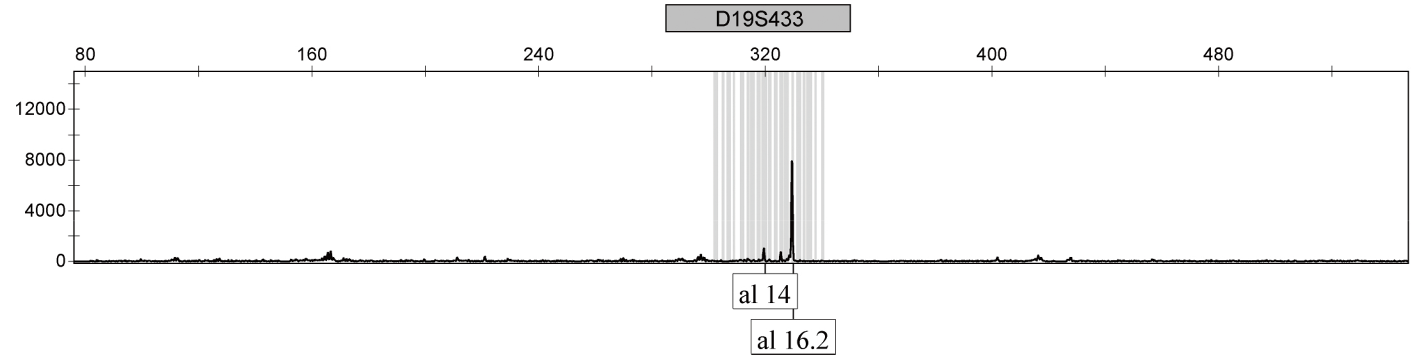

GeneMapper 4.0

CELL-13

| Sample File | Sample Name | Panel | OS | SQ |
|---|---|---|---|---|
| 92_D12_CellLineAuthentication-0516.fsa | OVCAR-3 | STR Profile 4-human | | |

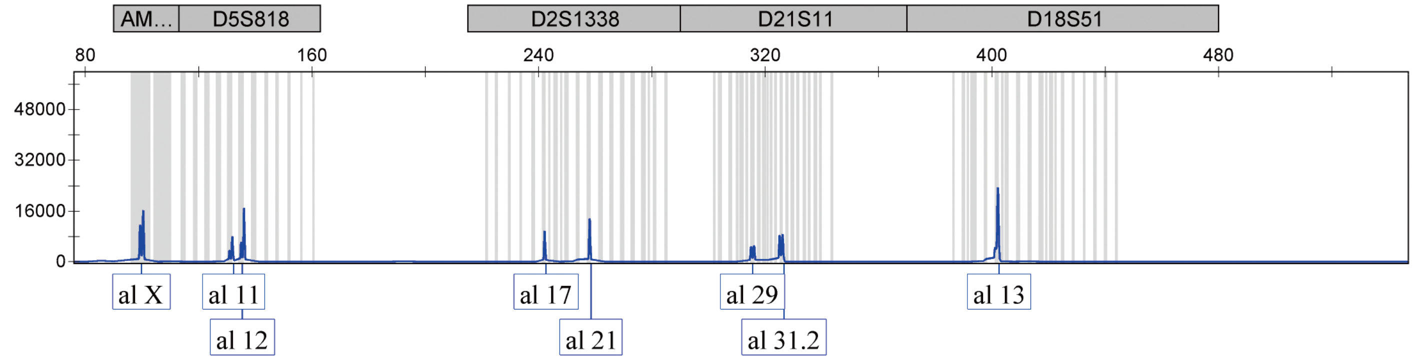


| | | | | |
|---|---|---|---|---|
| 92_D12_CellLineAuthentication-0516.fsa | OVCAR-3 | STR Profile 4-human | | |

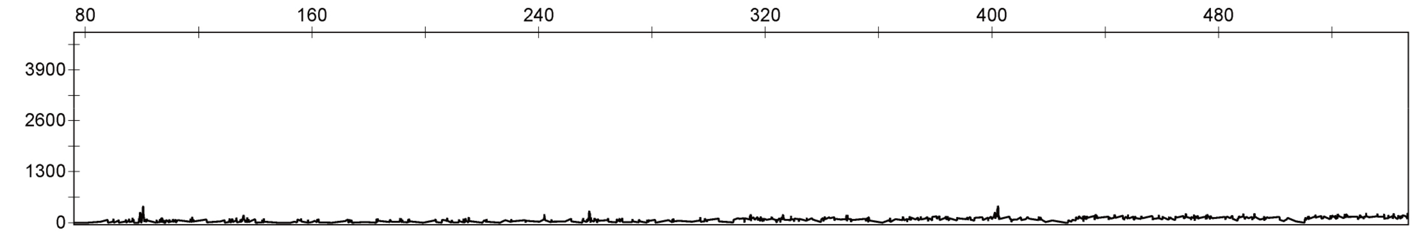

| Sample File | Sample Name | Panel | SQO | OS | SQ |
|---|---|---|---|---|---|
| 89_A12_CellLineAuthenaticon-1-1127.fsa | 293T | 21Plex_STR_Panel_v0.4 | | | |

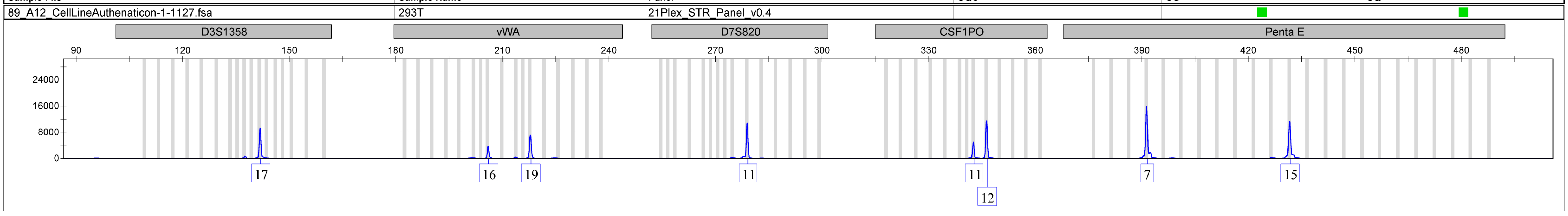


| | | | | | |
|---|---|---|---|---|---|
| 89_A12_CellLineAuthenaticon-1-1127.fsa | 293T | 21Plex_STR_Panel_v0.4 | | | |

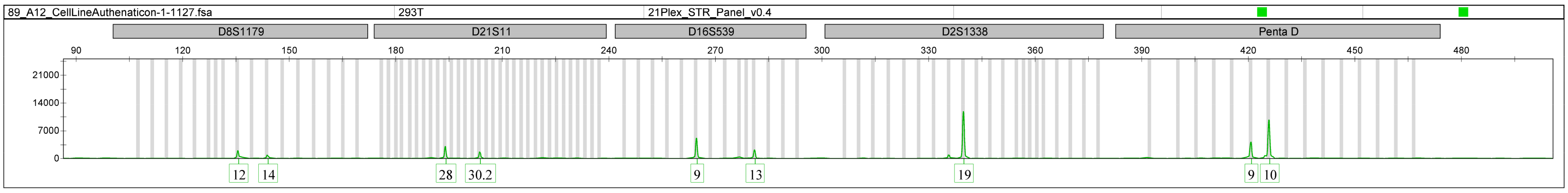


| | | | | | |
|---|---|---|---|---|---|
| 89_A12_CellLineAuthenaticon-1-1127.fsa | 293T | 21Plex_STR_Panel_v0.4 | | | |

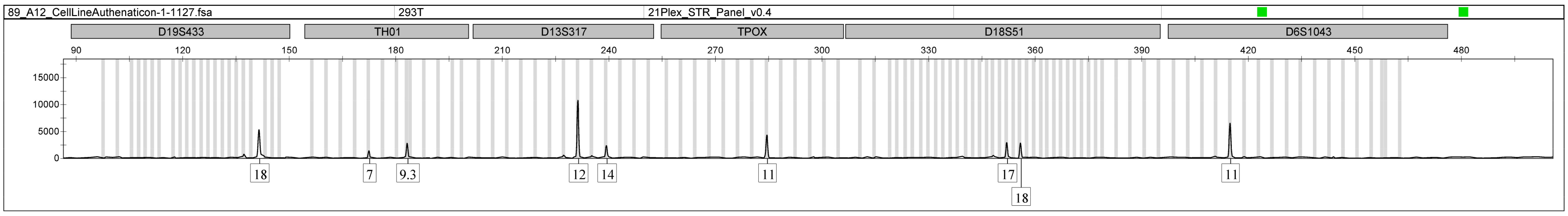


| | | | | | |
|---|---|---|---|---|---|
| 89_A12_CellLineAuthenaticon-1-1127.fsa | 293T | 21Plex_STR_Panel_v0.4 | | | |

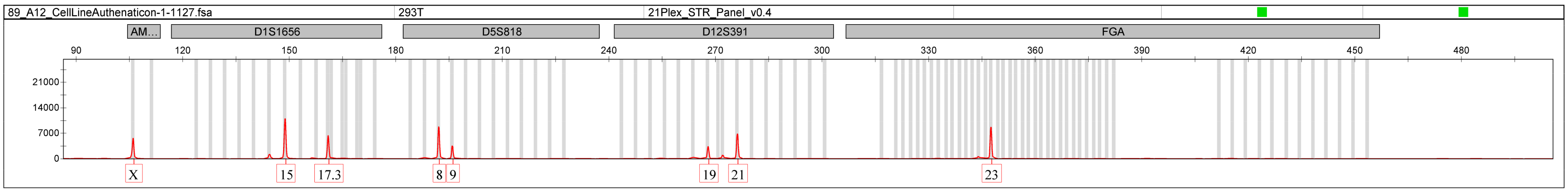

Supplement: Supplementary file 3 — STR profiling of cell lines [file 41419_2024_6816_MOESM3_ESM.pdf]
